# In-hospital inflammatory-nutritional-renal trajectory phenotypes and 90-day readmission or mortality in patients with acute decompensated heart failure: a retrospective cohort study

**DOI:** 10.3389/fcvm.2026.1900715

**Published:** 2026-07-31

**Authors:** Jianchao Liu, Hongtao Cai, Junbo Feng

**Affiliations:** 1 Department of Internal Medicine, Muchuan New District Hospital of Traditional Chinese Medicine, Leshan, Sichuan Province, China; 2 Department of Geriatric Medicine, The People’s Hospital of Changji Hui Autonomous Prefecture, Changji, Xinjiang Uygur Autonomous Region, China; 3 Department of Cardiology, Leshan Hospital of Traditional Chinese Medicine, Leshan, Sichuan Province, China

**Keywords:** acute decompensated heart failure, neutrophil-to-lymphocyte ratio, renal function, serum albumin, trajectory phenotype

## Abstract

**Background:**

Patients hospitalized with acute decompensated heart failure (ADHF) remain at high risk for early readmission and death after discharge. Static biomarkers may not fully capture the evolving inflammatory, nutritional, and renal status during hospitalization. We aimed to identify in-hospital inflammatory-nutritional-renal trajectory phenotypes and assess their associations with 90-day post-discharge outcomes.

**Methods:**

This single-center retrospective cohort study included 633 adults hospitalized with ADHF between January 2018 and December 2025 who survived to discharge and had serial measurements of neutrophil-to-lymphocyte ratio (NLR), serum albumin, and estimated glomerular filtration rate (eGFR) at admission, 48–72 h after admission, and before discharge. Multivariate latent class mixed modelling was used to identify trajectory phenotypes. The primary outcome was all-cause readmission or all-cause mortality within 90 days after discharge. Associations were evaluated using multivariable logistic regression, with supplementary time-to-event analyses.

**Results:**

Overall, 172 patients (27.2%) experienced the primary outcome. Three phenotypes were identified: Phenotype 1, low inflammation-stable nutrition-stable renal function (*n* = 348, 55.0%); Phenotype 2, moderate inflammation-declining albumin-stable renal function (*n* = 196, 31.0%); and Phenotype 3, high inflammation-low albumin-worsening renal function (*n* = 89, 14.0%). Event rates were 14.9%, 33.2%, and 61.8%, respectively. In the fully adjusted model, Phenotype 2 (odds ratio [OR], 2.12; 95% confidence interval [CI], 1.34–3.35) and Phenotype 3 (OR, 4.85; 95% CI, 2.58–9.12) were associated with the primary outcome. Additional adjustment for baseline NLR, albumin, and eGFR attenuated but did not eliminate these associations. The area under the receiver operating characteristic curve increased from 0.732 for the clinical model to 0.754 after adding baseline biomarkers and to 0.768 after further adding trajectory phenotype.

**Conclusions:**

In-hospital inflammatory-nutritional-renal trajectory phenotypes were associated with graded 90-day risks after ADHF discharge and provided limited additional prognostic information beyond baseline clinical and biomarker data. Prospective external validation is required before clinical implementation.

## 1 Introduction

Heart failure (HF) remains a major global health burden, and hospitalization for acute decompensated heart failure (ADHF) marks a period of substantial clinical instability and healthcare use (1). Patients hospitalized with HF are heterogeneous across ejection fraction phenotypes and commonly have multiple coexisting conditions, which complicates short-term risk assessment (2). Readmission and mortality remain frequent after discharge (3), and clinically relevant risk extends beyond the conventional 30-day window (4). In patients with acute HF, approximately one quarter may experience HF hospitalization or all-cause death within 90 days (5), underscoring the need for risk markers that reflect not only admission severity but also the patient's evolving condition during hospitalization.

Conventional ADHF risk assessment incorporates demographic characteristics, prior HF history, blood pressure, renal function, natriuretic peptides, left ventricular ejection fraction (LVEF) phenotype, and comorbidities. Although these variables are clinically informative, they are often evaluated at a single time point and may not adequately capture treatment response, persistent congestion, or residual vulnerability before discharge. Routinely available biomarkers can provide complementary prognostic information (6), but most studies have examined baseline values or isolated follow-up measurements. A trajectory-based approach may add value by incorporating both biomarker level and direction of change, thereby distinguishing patients with similar admission values but different in-hospital biological responses.

3The neutrophil-to-lymphocyte ratio (NLR), serum albumin, and estimated glomerular filtration rate (eGFR) represent complementary inflammatory, nutritional, and renal domains. Higher NLR has been associated with adverse HF outcomes (7), albumin-based indices reflect nutritional-inflammatory burden and prognosis (8), and renal function changes in ADHF may arise from congestion, hypoperfusion, diuretic response, or intrinsic kidney injury (9). Evaluating these markers jointly over time may therefore reveal multidimensional patterns that are not captured by any single static measurement. Although data-driven methods have identified clinically relevant HF subphenotypes (10), evidence remains limited regarding whether longitudinal trajectories of routinely available biomarkers can define reproducible ADHF phenotypes associated with early post-discharge risk. Therefore, this study aimed to identify in-hospital inflammatory-nutritional-renal trajectory phenotypes using serial NLR, serum albumin, and eGFR measurements and to evaluate their association with 90-day readmission or mortality among patients hospitalized with ADHF who survived to discharge.

## 2 Methods

### 2.1 Study design

This single-center retrospective cohort study evaluated the association between in-hospital inflammatory-nutritional-renal trajectory phenotypes and the 90-day composite risk of readmission or mortality among adults hospitalized with ADHF. Consecutive hospitalizations at Muchuan New District Hospital of Traditional Chinese Medicine from January 2018 to December 2025 were identified from the institutional electronic medical record system. The study was approved by the Institutional Review Board of Muchuan New District Hospital of Traditional Chinese Medicine (Approval No. MCZYY-IRB-2026-008), which waived the requirement for written informed consent because de-identified data generated during routine care were used. The study conformed to the Declaration of Helsinki and was reported according to the Strengthening the Reporting of Observational Studies in Epidemiology guidelines.

### 2.2 Study population

Potentially eligible admissions were screened using International Classification of Diseases, Tenth Revision heart failure codes and institution-specific diagnostic terms. ICD-10 coding was used only for initial screening. Two trained physicians independently reviewed the complete medical records using prespecified clinical criteria, and disagreements were resolved by consensus or adjudication by a senior cardiologist.

Patients were eligible if they were aged ≥18 years; had clinically confirmed ADHF as the principal reason for hospitalization according to contemporary European Society of Cardiology, American Heart Association/American College of Cardiology/Heart Failure Society of America, and Universal Definition criteria (11–13); had an available LVEF; had valid NLR, serum albumin, and eGFR measurements at all three prespecified clinical nodes; survived to discharge; and had ascertainable 90-day outcome status. ADHF required acute or worsening HF symptoms with objective evidence of pulmonary or systemic congestion, an underlying cardiac structural or functional abnormality, and urgent inpatient HF treatment.

Exclusion criteria were acute coronary syndrome, pulmonary embolism, sepsis or septic shock, acute severe valvular disease, severe anemia, or another acute condition constituting the principal reason for admission; maintenance dialysis; active malignancy, severe cirrhosis, nephrotic syndrome, active systemic inflammatory or autoimmune disease requiring chronic immunosuppression, organ transplantation, recent major surgery, severe trauma, or active major bleeding; length of stay <48 h; in-hospital death; substantial missingness of required baseline covariates; or unavailable 90-day outcomes. The source cohort for analyses addressing complete serial biomarker availability comprised 822 clinically eligible discharge survivors with ascertainable outcomes.

### 2.3 Data collection, variables, and definitions

Demographic, clinical, laboratory, echocardiographic, treatment, medication, and outcome data were extracted from the institutional electronic medical record, laboratory, echocardiography, physician-order, nursing, discharge, and follow-up systems. Baseline variables were defined using data obtained at admission or within the first 24 h unless otherwise specified.

Collected variables included age, sex, body mass index, prior HF hospitalization, smoking and alcohol status, vital signs, New York Heart Association functional class, signs of congestion, and major comorbidities. Comorbidity burden was summarized using the Charlson Comorbidity Index (14). Laboratory variables included complete blood count, renal and liver function indices, electrolytes, cardiac biomarkers, natriuretic peptides, and inflammatory markers. NLR was calculated as the absolute neutrophil count divided by the absolute lymphocyte count. eGFR was obtained from the laboratory system or calculated using the 2021 Chronic Kidney Disease Epidemiology Collaboration creatinine equation (15).

Echocardiographic variables included LVEF, left atrial diameter, left ventricular end-diastolic diameter, *E*/*e*′ ratio, valvular regurgitation, and estimated pulmonary artery systolic pressure. HF was classified as HFrEF, HFmrEF, or HFpEF according to contemporary guideline-defined LVEF categories (12).

In-hospital treatment variables included intravenous loop-diuretic dose, intravenous vasodilator use, inotrope or vasopressor use, ventilatory support, ultrafiltration, and ICU or coronary care unit care. Medication exposure before the pre-discharge measurement and discharge prescriptions were also recorded. Outcomes were ascertained through institutional readmission records, outpatient records, standardized telephone follow-up, and death registry information when available. Additional definitions and data-extraction procedures are provided in the Supplementary Material.

### 2.4 Trajectory variables and phenotype identification

Trajectory phenotypes were derived from NLR, serum albumin, and eGFR measured at three prespecified clinical nodes: T1, the first measurement within 0–24 h after admission; T2, a measurement obtained 48–72 h after admission; and T3, the final routine measurement obtained 24–48 h before discharge. For patients without testing during the predefined T3 window, the most recent measurement obtained after T2 and at least 72 h after admission was used. Patients without valid measurements at all three nodes were excluded from the primary trajectory analysis. A standardized hierarchical algorithm was used to select measurements when multiple eligible laboratory panels were available; full selection rules are reported in the Supplementary Material.

NLR was natural-log transformed, and log-transformed NLR, serum albumin, and eGFR were standardized as Z-scores. Multivariate latent class mixed models were fitted using the R package lcmm (16). Candidate models containing one to four latent classes were compared. The number of classes was selected using Bayesian information criterion as the primary criterion, supported by Akaike information criterion, sample-size-adjusted Bayesian information criterion, entropy, posterior class-membership probabilities, minimum class size, convergence, reproducibility, and clinical interpretability (17). Each patient was assigned to the class with the highest posterior probability. Detailed model specifications, initialization procedures, and convergence assessment are provided in the Supplementary Material.

### 2.5 Outcome definitions

The primary outcome was the composite of all-cause readmission or all-cause mortality within 90 days after discharge. All-cause readmission was defined as the first unplanned hospitalization for any reason; planned procedural, rehabilitation, or routine evaluation admissions were excluded. Discharge served as time zero.

The principal disease-specific secondary outcome was HF-related readmission or all-cause mortality within 90 days. Other secondary outcomes included 90-day HF-related readmission, all-cause mortality, cardiovascular mortality, 30-day and 180-day all-cause readmission or mortality, ICU or coronary care unit admission or transfer during the index hospitalization, prolonged length of stay, inadequate pre-discharge NT-proBNP reduction, and in-hospital worsening renal function. HF-related readmissions were independently adjudicated by two physicians. Prolonged length of stay was defined as a duration above the cohort's 75th percentile. Worsening renal function was defined as an increase in serum creatinine of ≥0.3 mg/dL within 48 h or to ≥1.5 times baseline within 7 days, according to the creatinine component of the Kidney Disease: Improving Global Outcomes acute kidney injury criteria (18).

### 2.6 Sample size calculation

Sample size considerations addressed both latent-class identification and multivariable outcome analysis. Based on trajectory-modelling guidance recommending that the smallest class contain at least 5% of the cohort, approximately 400–500 patients were considered necessary to identify up to three stable phenotypes (19).

The outcome model was expected to include approximately 8–10 principal adjustment covariates. Using an events-per-variable range of 10–15, approximately 100–150 primary outcome events were required. Previous acute HF studies reported a 90-day HF hospitalization or mortality rate of approximately 25% (5) and a 90-day readmission rate of 31.2% (4). Assuming an event rate of 20%–30%, the target analytic sample was therefore 500–750 patients.

### 2.7 Statistical analysis

Analyses were performed using R version 4.5.1. Normally distributed continuous variables were summarized as mean ± standard deviation and compared using one-way analysis of variance. Non-normally distributed variables were presented as median and interquartile range and compared using the Kruskal–Wallis test. Categorical variables were summarized as *n* (%) and compared using the *χ*^2^ test or Fisher's exact test. All tests were two-sided, with *p* < 0.05 considered statistically significant.

Patients without primary outcome data or complete T1–T3 trajectory measurements were excluded from the primary analysis. Covariates with >20% missingness were not entered into the main models. Variables with 5%–20% missingness were imputed using multiple imputation by chained equations with 20 datasets; complete-case analysis was used when missingness was <5%. Additional imputation details are provided in the Supplementary Material.

Associations between trajectory phenotype and the primary outcome were assessed using logistic regression. Model 1 was unadjusted. Model 2 adjusted for age, sex, body mass index, previous HF hospitalization, LVEF phenotype, and New York Heart Association class. Model 3 additionally adjusted for diabetes, coronary artery disease, atrial fibrillation, chronic kidney disease, admission systolic blood pressure, baseline log-transformed NT-proBNP, serum sodium, and discharge SGLT2 inhibitor, renin–angiotensin system inhibitor, beta-blocker, and mineralocorticoid receptor antagonist use. An additional sensitivity model included baseline log-transformed NLR, serum albumin, and eGFR. Cox proportional hazards models were used as supplementary time-to-event analyses when event dates were available. HF-related readmission was additionally evaluated using a Fine–Gray model with death as a competing event.

Internal stability of the selected trajectory solution was assessed using 500 patient-level bootstrap samples in which the complete class-selection procedure was repeated. Stability was summarized by the frequency of selecting the original class solution, class proportions, and adjusted Rand index. Detailed bootstrap procedures are reported in the Supplementary Material.

Incremental prognostic value was evaluated using three nested logistic models: a clinical model containing age, sex, LVEF phenotype, New York Heart Association class, previous HF hospitalization, chronic kidney disease, admission systolic blood pressure, and baseline log-transformed NT-proBNP; a clinical-plus-baseline-biomarker model additionally containing baseline log-transformed NLR, serum albumin, and eGFR; and a trajectory-enhanced model further containing trajectory phenotype. Model performance was compared using area under the receiver operating characteristic curve, DeLong tests, likelihood-ratio tests, Brier scores, calibration plots, and decision-curve analysis. These analyses assessed incremental prognostic information and were not intended as formal prediction-model development or external validation.

Sensitivity analyses examined alternative missing-data approaches, an HF-specific composite outcome, exclusion of patients with baseline eGFR <30 mL/min/1.73 m^2^, an alternative T3 window, substitution of serum creatinine for eGFR, inverse probability-of-observation weighting, T1–T2-only trajectory classification, and additional adjustment for treatment intensity, NT-proBNP response, pre-T3 medication exposure, and baseline trajectory biomarkers. Full specifications of these analyses are provided in the Supplementary Material.

## 3 Results

### 3.1 Study population and baseline characteristics

**Figure 1:**
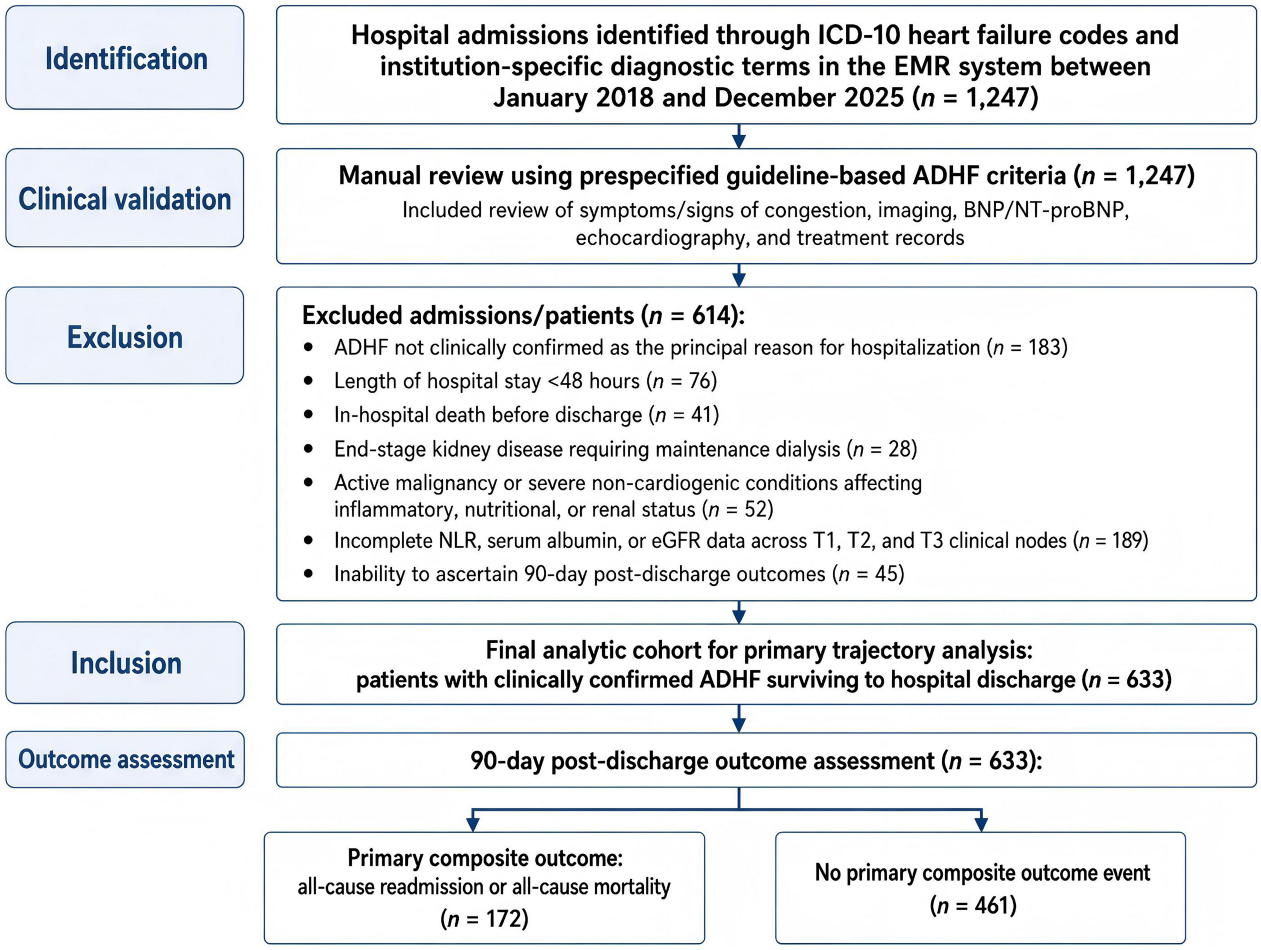
Flow diagram of patient enrollment.

Among 1,247 admissions identified by the initial ICD-10-based heart failure query, all potentially eligible records underwent manual clinical validation using the prespecified ADHF criteria. Overall, 822 clinically eligible patients survived to discharge and had ascertainable 90-day outcomes. Of these, 633 patients (77.0%) had valid NLR, serum albumin, and eGFR measurements at all three prespecified nodes and constituted the primary trajectory cohort, whereas 189 patients lacked a complete three-node biomarker profile. Within the primary trajectory cohort, 172 patients (27.2%) experienced the primary composite outcome (Figure 1). Baseline characteristics stratified by the primary outcome are summarized in Table 1. Compared with the non-event group, patients experiencing the composite outcome were significantly older with a higher comorbidity burden (CCI), including greater prevalences of prior HF hospitalization, chronic kidney disease, atrial fibrillation, and NYHA class IV symptoms (all *p* < 0.05). Furthermore, the event group exhibited higher baseline NLR, serum creatinine, BUN, uric acid, hs-cTnT, and log-transformed NT-proBNP, but lower SBP, DBP, hemoglobin, serum sodium, serum albumin, and eGFR (all *p* < 0.05). LVEF phenotype distribution also differed significantly (*p* = 0.044). Discharge beta-blocker use was less frequent in the event group than in the non-event group (66.3% vs. 75.5%; *p* = 0.022), whereas the other reported discharge medications, including SGLT2 inhibitors, ARNI, ACEI/ARB, and MRA, did not differ significantly between groups.

**Table 1:** Baseline clinical characteristics according to 90-day readmission or mortality.

Variables	Total (*N* = 633)	Non-event Group (*n* = 461)	Event Group (*n* = 172)	*P*-value
Demographics and clinical presentation				
Age, years, mean ± SD	70.9 ± 11.0	69.4 ± 11.2	74.8 ± 9.3	<0.001
Male sex, *n* (%)	358 (56.6)	265 (57.5)	93 (54.1)	0.434
BMI, kg/m^2^, mean ± SD	24.3 ± 4.2	24.5 ± 4.1	23.9 ± 4.3	0.117
Admission source: Emergency department, *n* (%)	412 (65.1)	291 (63.1)	121 (70.3)	0.094
Current or former smoking, *n* (%)	285 (45.0)	205 (44.5)	80 (46.5)	0.648
Alcohol consumption, *n* (%)	179 (28.3)	134 (29.1)	45 (26.2)	0.469
CCI, median (IQR)	2 (1, 4)	2 (1, 4)	3 (2, 5)	<0.001
NYHA functional class, *n* (%)				<0.001
Class II	98 (15.5)	83 (18.0)	15 (8.7)	
Class III	359 (56.7)	269 (58.4)	90 (52.3)	
Class IV	176 (27.8)	109 (23.6)	67 (38.4)	
CCU admission, *n* (%)	114 (18.0)	76 (16.5)	38 (22.1)	0.108
SBP, mmHg, mean ± SD	124.2 ± 19.5	126.3 ± 19.8	118.6 ± 17.4	<0.001
DBP, mmHg, mean ± SD	75.6 ± 12.4	76.2 ± 12.5	73.9 ± 11.8	0.041
HR, beats/min, mean ± SD	88.4 ± 16.2	87.9 ± 16.1	89.7 ± 16.5	0.224
Comorbidities, *n* (%)				
Prior HF hospitalization	229 (36.2)	145 (31.5)	84 (48.8)	<0.001
Hypertension	421 (66.5)	305 (66.2)	116 (67.4)	0.767
Diabetes mellitus	264 (41.7)	186 (40.3)	78 (45.3)	0.262
Coronary artery disease	315 (49.8)	225 (48.8)	90 (52.3)	0.445
Prior myocardial infarction	134 (21.2)	92 (20.0)	42 (24.4)	0.232
Atrial fibrillation	246 (38.9)	165 (35.8)	81 (47.1)	0.010
Stroke	102 (16.1)	71 (15.4)	31 (18.0)	0.432
Chronic kidney disease	196 (31.0)	122 (26.5)	74 (43.0)	<0.001
Chronic obstructive pulmonary disease	128 (20.2)	91 (19.7)	37 (21.5)	0.621
Anemia	167 (26.4)	114 (24.7)	53 (30.8)	0.129
Echocardiographic parameters				
LVEF phenotype, *n* (%)				0.044
HFrEF (≤40%)	241 (38.1)	165 (35.8)	76 (44.2)	
HFmrEF (41–49%)	136 (21.5)	104 (22.6)	32 (18.6)	
HFpEF (≥50%)	256 (40.4)	192 (41.6)	64 (37.2)	
LAD, mm, mean ± SD	45.3 ± 8.2	44.8 ± 8.1	46.7 ± 8.4	0.012
*E*/*e*′ ratio, median (IQR)	14.8 (11.2, 18.6)	14.3 (10.9, 17.8)	16.1 (12.4, 20.2)	0.004
Baseline laboratory parameters				
Hemoglobin, g/L, mean ± SD	122.4 ± 21.6	123.8 ± 21.2	118.6 ± 22.4	0.009
NLR, median (IQR)	4.3 (2.8, 6.7)	3.9 (2.5, 5.7)	6.2 (4.1, 9.8)	<0.001
Serum albumin, g/L, mean ± SD	35.5 ± 4.4	36.2 ± 4.1	33.8 ± 4.5	<0.001
eGFR, mL/min/1.73 m^2^, mean ± SD	62.1 ± 21.5	65.8 ± 21.3	52.4 ± 18.6	<0.001
Serum creatinine, μmol/L, median (IQR)	108 (82, 145)	101 (78, 131)	132 (95, 176)	<0.001
BUN, mmol/L, median (IQR)	8.4 (6.1, 12.3)	7.9 (5.8, 11.2)	10.3 (7.3, 14.8)	<0.001
Sodium, mmol/L, mean ± SD	138.4 ± 4.8	138.8 ± 4.6	137.3 ± 5.2	0.001
Uric acid, μmol/L, mean ± SD	412 ± 134	398 ± 128	449 ± 142	<0.001
log(NT-proBNP), mean ± SD	8.30 ± 1.13	8.14 ± 1.12	8.72 ± 1.05	<0.001
hs-cTnT, ng/L, median (IQR)	28 (15, 56)	25 (13, 50)	36 (20, 71)	<0.001
Discharge medications, *n* (%)				
SGLT2i	208 (32.9)	158 (34.3)	50 (29.1)	0.223
ARNI	241 (38.1)	182 (39.5)	59 (34.3)	0.236
ACEI/ARB	165 (26.1)	125 (27.1)	40 (23.3)	0.332
Beta-blockers	462 (73.0)	348 (75.5)	114 (66.3)	0.022
MRA	385 (60.8)	288 (62.5)	97 (56.4)	0.167
Loop diuretics	538 (85.0)	386 (83.7)	152 (88.4)	0.146

Data are presented as mean ± SD, median (IQR), or *n* (%). eGFR was calculated using the 2021 CKD-EPI creatinine equation. NT-proBNP was natural-log transformed prior to statistical comparison due to positive skewness. BMI, body mass index; CCI, Charlson Comorbidity Index; NYHA, New York Heart Association; CCU, coronary care unit; SBP, systolic blood pressure; DBP, diastolic blood pressure; HR, heart rate; HF, heart failure; LVEF, left ventricular ejection fraction; HFrEF, HF with reduced ejection fraction; HFmrEF, HF with mildly reduced ejection fraction; HFpEF, HF with preserved ejection fraction; LAD, left atrial diameter; NLR, neutrophil-to-lymphocyte ratio; eGFR, estimated glomerular filtration rate; BUN, blood urea nitrogen; NT-proBNP, N-terminal pro-B-type natriuretic peptide; hs-cTnT, high-sensitivity cardiac troponin T; SGLT2i, sodium-glucose cotransporter 2 inhibitor; ARNI, angiotensin receptor-neprilysin inhibitor; ACEI, angiotensin-converting enzyme inhibitor; ARB, angiotensin II receptor blocker; MRA, mineralocorticoid receptor antagonist.

### 3.2 Dynamic changes in inflammatory, nutritional, and renal biomarkers during early hospitalization

**Figure 2:**
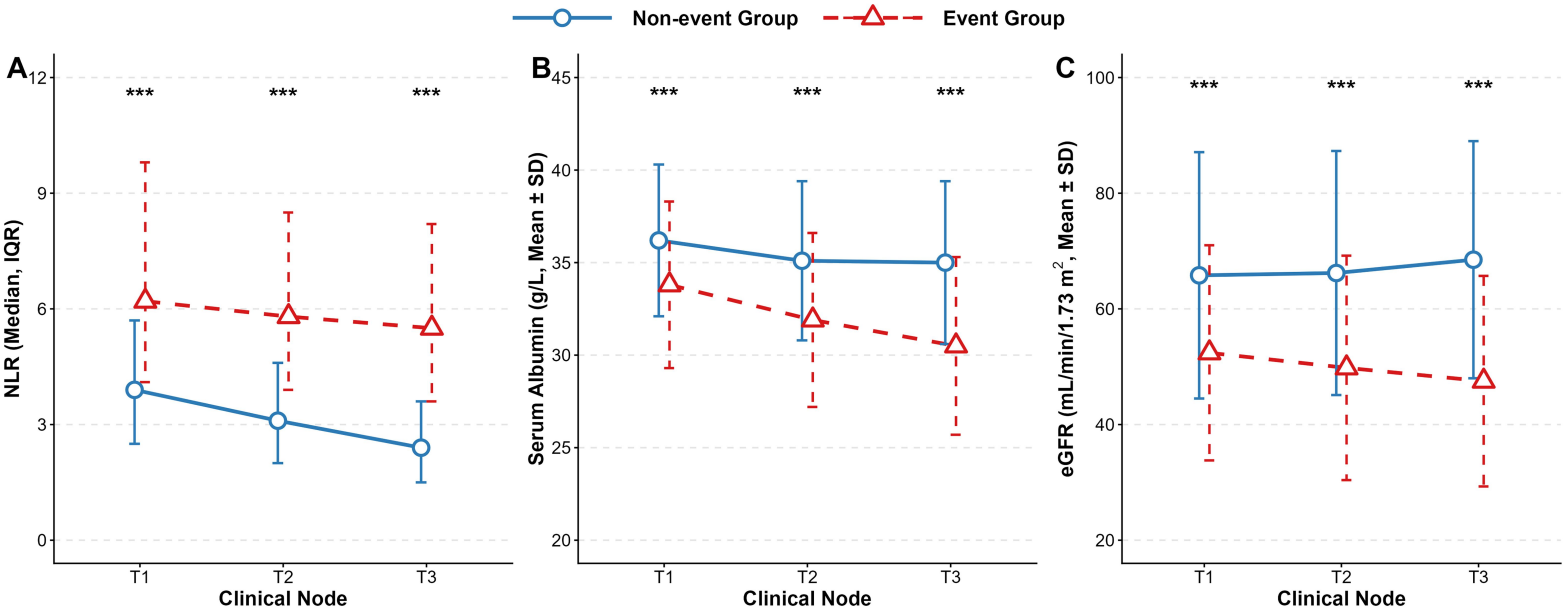
Temporal patterns of NLR, serum albumin, and eGFR during hospitalization. Three multi-panel line graphs display the temporal trajectories of **(A)** neutrophil-to-lymphocyte ratio (NLR), **(B)** serum albumin, and **(C)** estimated glomerular filtration rate (eGFR) across the T1 (admission), T2 (early-treatment), and T3 (pre-discharge) clinical nodes. Solid blue lines with circles denote the non-event group, whereas dashed red lines with triangles denote the event group (patients experiencing 90-day readmission or mortality). Data are presented as median with interquartile ranges (for NLR) or mean ± standard deviation (for serum albumin and eGFR). Statistical significance between the event and non-event groups at each time node is indicated: ***p < 0.001.

During the early hospitalization period across the predefined T1, T2, and T3 clinical nodes, distinct dynamic alterations in core biomarkers were observed. The median collection time for the T2 early-treatment node was 59.6 h after admission (IQR, 54.8–65.2 h). All three core biomarkers were obtained from the same routine phlebotomy episode in 609 patients (96.2%); in the remaining 24 patients (3.8%), the measurements were obtained within the prespecified maximum interval of 6 h. In the overall cohort, NLR generally decreased as decongestive and medical therapies progressed, serum albumin exhibited a mild initial decline from T1 to T2 followed by stabilization at T3, and eGFR remained relatively stable with minor aggregate fluctuations. Log-transformed NT-proBNP demonstrated a consistent downward trajectory across the three nodes in the overall population. When stratified by the occurrence of the 90-day composite outcome, differences between the event and non-event groups were observed at each clinical node for the evaluated biomarkers (Table 2, Figure 2). Compared with the non-event group, patients who experienced 90-day readmission or mortality maintained persistently higher NLR and lower serum albumin and eGFR across all three clinical nodes (all *p* < 0.001). Notably, while the non-event group demonstrated a progressive decline in NLR and an increase in eGFR by the T3 node, the event group exhibited a blunted reduction in NLR coupled with a progressive decline in eGFR from T1 to T3. Similarly, serum albumin levels in the event group showed a more pronounced and sustained decrease throughout the hospitalization compared to the relatively stable trend observed in the non-event group. Other biomarkers, including BUN and serum creatinine, remained persistently elevated in the event group across all time points, whereas serum sodium levels were consistently lower and demonstrated limited recovery by the pre-discharge node (all *p* < 0.05).

**Table 2:** Dynamic changes in inflammatory, nutritional, and renal biomarkers during hospitalization.

Biomarker and Node	Total (*N* = 633)	Non-event Group (*n* = 461)	Event Group (*n* = 172)	*P*-value
NLR, median (IQR)				
T1	4.3 (2.8, 6.7)	3.9 (2.5, 5.7)	6.2 (4.1, 9.8)	<0.001
T2	3.5 (2.2, 5.4)	3.1 (2.0, 4.6)	5.8 (3.9, 8.5)	<0.001
T3	2.9 (1.8, 4.5)	2.4 (1.5, 3.6)	5.5 (3.6, 8.2)	<0.001
Serum albumin, g/L, mean ± SD				
T1	35.5 ± 4.4	36.2 ± 4.1	33.8 ± 4.5	<0.001
T2	34.2 ± 4.6	35.1 ± 4.3	31.9 ± 4.7	<0.001
T3	34.0 ± 4.7	35.0 ± 4.4	30.5 ± 4.8	<0.001
eGFR, mL/min/1.73 m^2^, mean ± SD				
T1	62.1 ± 21.5	65.8 ± 21.3	52.4 ± 18.6	<0.001
T2	61.5 ± 22.0	66.2 ± 21.1	49.8 ± 19.4	<0.001
T3	62.8 ± 21.8	68.5 ± 20.5	47.5 ± 18.2	<0.001
Serum creatinine, μmol/L, median (IQR)				
T1	108 (82, 145)	101 (78, 131)	132 (95, 176)	<0.001
T2	110 (84, 150)	100 (77, 130)	141 (102, 188)	<0.001
T3	105 (80, 142)	95 (74, 122)	148 (108, 196)	<0.001
BUN, mmol/L, median (IQR)				
T1	8.4 (6.1, 12.3)	7.9 (5.8, 11.2)	10.3 (7.3, 14.8)	<0.001
T2	8.8 (6.3, 13.0)	8.1 (5.9, 11.5)	11.2 (8.1, 16.2)	<0.001
T3	8.2 (5.9, 12.0)	7.4 (5.5, 10.4)	12.0 (8.8, 17.5)	<0.001
Sodium, mmol/L, mean ± SD				
T1	138.4 ± 4.8	138.8 ± 4.6	137.3 ± 5.2	0.001
T2	138.9 ± 4.5	139.4 ± 4.3	137.5 ± 4.9	<0.001
T3	139.5 ± 4.2	140.2 ± 3.9	137.6 ± 4.8	<0.001
log(NT-proBNP), mean ± SD				
T1	8.30 ± 1.13	8.14 ± 1.12	8.72 ± 1.05	<0.001
T2	7.95 ± 1.20	7.70 ± 1.15	8.55 ± 1.10	<0.001
T3	7.42 ± 1.25	7.10 ± 1.18	8.38 ± 1.08	<0.001

Data are presented as mean ± SD or median (IQR). T1, admission node, defined as the first available measurement within 0–24 h after admission; T2, early-treatment node, defined as the complete laboratory panel obtained within 48–72 h and closest to 60 h after admission; T3, pre-discharge node, defined as the last routine assessment within 24–48 h before discharge. For T2, measurements from the same phlebotomy episode were preferentially used; otherwise, component measurements were required to occur within 6 h.

### 3.3 Identification of inflammatory-nutritional-renal trajectory phenotypes

A multivariate LCMM identified distinct trajectory phenotypes based on longitudinal changes in log-transformed NLR, serum albumin, and eGFR (Table 3). Among the one- to four-class candidates, the three-class solution was selected according to the prespecified hierarchical criteria. It had the lowest BIC (8,000.7), adequate entropy (0.84), mean posterior class-membership probabilities ranging from 0.82 to 0.89, and no class containing <5% of the cohort. Although the four-class solution had lower AIC and sample-size-adjusted BIC values, its BIC increased to 8,012.5, entropy decreased to 0.76, and the additional class contained only 4.2% of patients without a clearly distinct trajectory pattern. The retained three-class solution also demonstrated satisfactory convergence: 93 of the 100 randomized initial-value runs converged to the same maximum log-likelihood, whereas seven converged to inferior local solutions.

**Table 3:** Model fit indices for inflammatory-nutritional-renal trajectory classification.

Number of classes	Log-likelihood	AIC	BIC	saBIC	Entropy	Minimum class size (%)	Mean posterior probability
1	−4,500.1	9,012.2	9,038.9	9,019.8	—	100.0	1.00
2	−4,100.5	8,223.0	8,267.5	8,235.6	0.79	35.5	0.85–0.88
3	−3,950.2	7,938.4	8,000.7	7,956.1	0.84	14.0	0.82–0.89
4	−3,910.8	7,875.6	8,012.5	7,900.2	0.76	4.2	0.71–0.81

BIC was the primary information criterion for class-number selection, while AIC and saBIC were considered supportive. Classification quality, minimum class size, reproducibility across randomized initial values, and clinical distinctiveness of the trajectory patterns were also considered. The three-class solution was retained because it had the lowest BIC, adequate entropy and posterior classification probabilities, and no class containing <5% of the cohort. Although the four-class solution had lower AIC and saBIC values, it was rejected because it had a higher BIC, lower entropy, and a sparse additional class comprising 4.2% of patients without a clearly distinct trajectory pattern. The four-class solution was rejected despite its lower AIC because it had a higher BIC, lower entropy, and a sparse additional class comprising 4.2% of patients. AIC, Akaike Information Criterion; BIC, Bayesian Information Criterion; saBIC, sample-size-adjusted Bayesian Information Criterion.

Bootstrap internal validation supported the stability of the selected solution (Supplementary Table S1). The three-class model was reselected in 438 of 500 bootstrap samples (87.6%); two- and four-class solutions were selected in 8.2% and 4.2%, respectively. Among bootstrap samples retaining three classes, the median adjusted Rand index for agreement with the original classification was 0.81 (IQR, 0.74–0.88). Median bootstrap class proportions were 54.7% (IQR, 52.1%–57.4%), 31.2% (IQR, 28.8%–33.6%), and 14.1% (IQR, 12.2%–16.0%), closely corresponding to the original proportions of 55.0%, 31.0%, and 14.0%.

**Figure 3:**
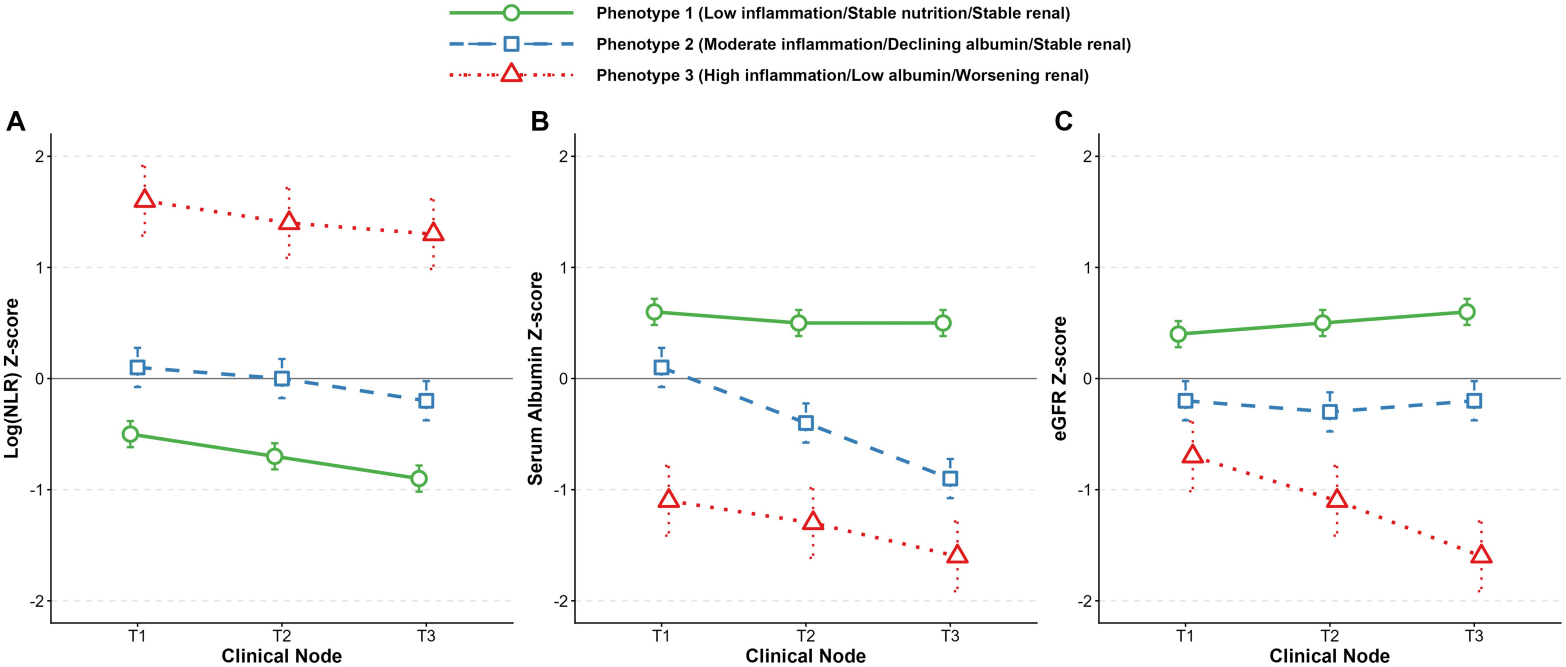
In-hospital inflammatory-nutritional-renal trajectory phenotypes in patients with acute decompensated heart failure. Three multi-panel line graphs illustrate the distinct temporal trajectories of **(A)** log-transformed neutrophil-to-lymphocyte ratio (NLR), **(B)** serum albumin, and **(C)** estimated glomerular filtration rate (eGFR) across the T1 (admission), T2 (early-treatment), and T3 (pre-discharge) clinical nodes. The *Y*-axis represents the standardized values (Z-scores) of each biomarker to allow commensurate visualization across different measurement scales. The solid grey horizontal line at 0 represents the mean standardized level of the overall study cohort. Three distinct trajectory classes are depicted: Phenotype 1 (low inflammation-stable nutrition-stable renal) is indicated by the solid green line with circles; Phenotype 2 (moderate inflammation-declining albumin-stable renal) by the dashed blue line with squares; and Phenotype 3 (high inflammation-low albumin-worsening renal) by the dotted red line with triangles. Data points and error bars represent the mean standardized values and their 95% confidence intervals.

Based on biomarker temporal patterns (Figure 3), three phenotypes were characterized: Phenotype 1 (“low inflammation-stable nutrition-stable renal”; *n* = 348, 55.0%), maintaining the lowest NLR and highest stable serum albumin and eGFR; Phenotype 2 (“moderate inflammation-declining albumin-stable renal”; *n* = 196, 31.0%), exhibiting intermediate NLR, progressive albumin decline, and preserved eGFR; and Phenotype 3 (“high inflammation-low albumin-worsening renal”; *n* = 89, 14.0%), defined by persistently elevated NLR, consistently low albumin, and continuously declining eGFR.

Baseline characteristics across phenotypes are detailed in Table 4. A prominent clinical risk gradient emerged: compared with Phenotypes 1 and 2, patients in Phenotype 3 were significantly older with the highest CCI, including greater prevalences of prior HF hospitalization, chronic kidney disease, atrial fibrillation, and NYHA class IV symptoms (all *p* < 0.05). Furthermore, Phenotype 3 exhibited a disproportionate prevalence of HFrEF and the highest baseline levels of serum creatinine, BUN, uric acid, and log-transformed NT-proBNP, alongside the lowest admission SBP, hemoglobin, and serum sodium (all *p* < 0.001). Remaining demographics, admission sources, and diabetes prevalence were comparable across groups (*p* > 0.05). In-hospital treatment intensity differed across trajectory phenotypes (Supplementary Table S2). Phenotype 3 received a higher cumulative intravenous loop-diuretic dose and more frequently required inotropes or vasopressors, non-invasive ventilation, and ICU/CCU care. In contrast, exposure before T3 to SGLT2 inhibitors, GLP-1RAs, and systemic corticosteroids did not differ significantly across phenotypes. No included patient received other systemic immunosuppressive therapy during the trajectory window.

**Table 4:** Baseline characteristics across trajectory phenotypes.

Variables	Phenotype 1 (*n* = 348)	Phenotype 2 (*n* = 196)	Phenotype 3 (*n* = 89)	*P*-value
Age, years, mean ± SD	68.5 ± 11.4	72.1 ± 10.1	77.6 ± 8.8	<0.001
Male sex, *n* (%)	195 (56.0)	114 (58.2)	49 (55.1)	0.824
BMI, kg/m^2^, mean ± SD	24.5 ± 4.1	24.2 ± 4.3	23.8 ± 4.4	0.317
Admission source: ED, *n* (%)	225 (64.7)	129 (65.8)	58 (65.2)	0.967
Current or former smoking, *n* (%)	154 (44.3)	90 (45.9)	41 (46.1)	0.915
CCI, median (IQR)	2 (1, 3)	3 (2, 4)	4 (2, 5)	<0.001
NYHA functional class, *n* (%)				<0.001
Class II	68 (19.5)	23 (11.7)	7 (7.9)	
Class III	208 (59.8)	114 (58.2)	37 (41.6)	
Class IV	72 (20.7)	59 (30.1)	45 (50.6)	
SBP, mmHg, mean ± SD	127.8 ± 19.3	122.5 ± 18.5	113.8 ± 17.6	<0.001
Comorbidities, *n* (%)				
Prior HF hospitalization	90 (25.9)	85 (43.4)	54 (60.7)	<0.001
Diabetes mellitus	143 (41.1)	81 (41.3)	40 (44.9)	0.795
Atrial fibrillation	121 (34.8)	77 (39.3)	48 (53.9)	0.004
Chronic kidney disease	62 (17.8)	70 (35.7)	64 (71.9)	<0.001
Echocardiographic parameters				
LVEF phenotype, *n* (%)				<0.001
HFrEF (≤40%)	110 (31.6)	76 (38.8)	55 (61.8)	
HFmrEF (41–49%)	78 (22.4)	43 (21.9)	15 (16.9)	
HFpEF (≥50%)	160 (46.0)	77 (39.3)	19 (21.3)	
Baseline laboratory parameters				
Hemoglobin, g/L, mean ± SD	125.6 ± 20.8	120.4 ± 21.5	114.3 ± 22.1	<0.001
NLR, median (IQR)	3.2 (2.2, 4.8)	4.9 (3.4, 7.1)	8.7 (5.8, 12.4)	<0.001
Serum albumin, g/L, mean ± SD	37.2 ± 3.8	34.3 ± 4.1	31.5 ± 4.4	<0.001
eGFR, mL/min/1.73 m^2^, mean ± SD	70.5 ± 19.8	55.2 ± 18.5	44.4 ± 17.2	<0.001
Serum creatinine, μmol/L, median (IQR)	96 (75, 125)	110 (84, 152)	155 (112, 198)	<0.001
BUN, mmol/L, median (IQR)	7.3 (5.4, 10.1)	9.2 (6.8, 13.5)	12.8 (9.1, 18.2)	<0.001
Sodium, mmol/L, mean ± SD	139.1 ± 4.5	138.2 ± 4.9	136.1 ± 5.3	<0.001
Uric acid, μmol/L, mean ± SD	395 ± 120	415 ± 135	472 ± 155	<0.001
log (NT-proBNP), mean ± SD	8.05 ± 1.10	8.42 ± 1.08	9.02 ± 0.95	<0.001

Data are presented as mean ± SD, median (IQR), or *n* (%). eGFR was calculated using the 2021 CKD-EPI creatinine equation. ED, emergency department; CCI, Charlson Comorbidity Index; NYHA, New York Heart Association; SBP, systolic blood pressure; HF, heart failure; LVEF, left ventricular ejection fraction; HFrEF, HF with reduced ejection fraction; HFmrEF, HF with mildly reduced ejection fraction; HFpEF, HF with preserved ejection fraction; NLR, neutrophil-to-lymphocyte ratio; eGFR, estimated glomerular filtration rate; BUN, blood urea nitrogen; NT-proBNP, N-terminal pro-B-type natriuretic peptide.

**Figure 4:**
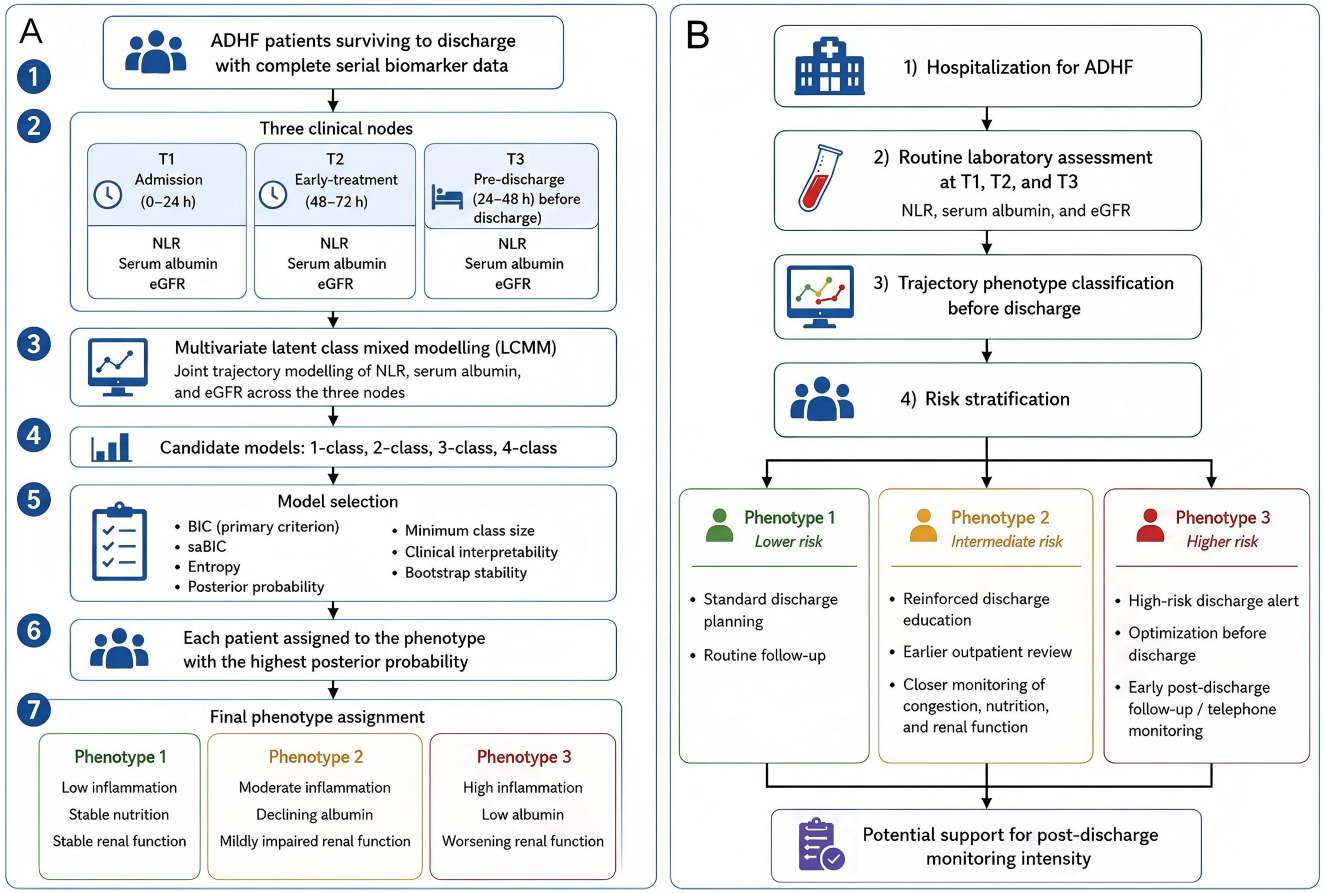
Schematic illustration of trajectory modelling, phenotype assignment, and potential clinical application. **(A)** Overview of the trajectory modelling and phenotype assignment process. Patients with acute decompensated heart failure (ADHF) who survived to discharge and had complete serial biomarker data were evaluated at three prespecified clinical nodes: T1 (admission, 0–24 h), T2 (early treatment, 48–72 h), and T3 (pre-discharge, 24–48 h before discharge). Serial measurements of neutrophil-to-lymphocyte ratio (NLR), serum albumin, and estimated glomerular filtration rate (eGFR) were jointly entered into a multivariate latent class mixed model (LCMM). Candidate one- to four-class solutions were compared, and the final phenotype assignment was based on prespecified model-selection criteria and the highest posterior probability. **(B)** Conceptual illustration of the potential clinical application of the identified trajectory phenotypes. Serial routine laboratory assessment during hospitalization may support phenotype-based risk stratification before discharge and may help identify patients requiring different intensities of post-discharge monitoring. This panel is intended to illustrate potential translational relevance and does not imply that the phenotype classification has been prospectively validated for clinical decision-making. ADHF, acute decompensated heart failure; NLR, neutrophil-to-lymphocyte ratio; eGFR, estimated glomerular filtration rate; LCMM, latent class mixed model; BIC, Bayesian information criterion; saBIC, sample-size-adjusted Bayesian information criterion.

A schematic overview of the trajectory modelling process, phenotype assignment, and potential clinical application is shown in Figure 4.

### 3.4 Clinical outcomes across trajectory phenotypes

**Figure 5:**
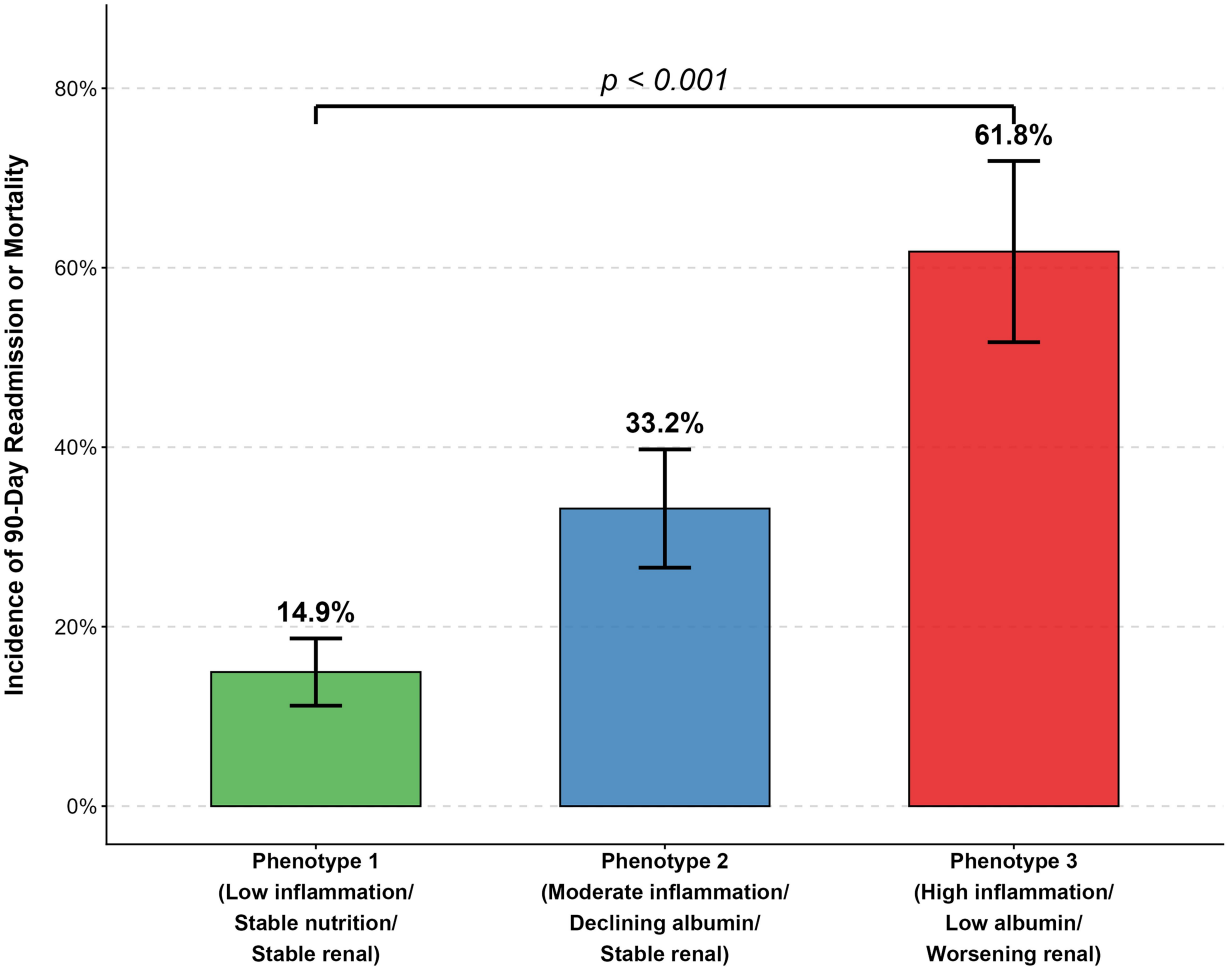
Incidence of 90-day readmission or mortality across trajectory phenotypes. A bar chart illustrates the incidence rates of the primary composite outcome (90-day all-cause readmission or mortality) stratified by the three distinct trajectory phenotypes. The *X*-axis represents the three trajectory groups: Phenotype 1 (low inflammation-stable nutrition-stable renal), Phenotype 2 (moderate inflammation-declining albumin-stable renal), and Phenotype 3 (high inflammation-low albumin-worsening renal). The *Y*-axis represents the event incidence rate (%). The height of each bar corresponds to the specific incidence rate for each phenotype, with error bars denoting the 95% confidence intervals of the proportions. Statistical significance across the groups is marked (*p* < 0.001).

The incidence of the primary and secondary clinical outcomes across the three distinct trajectory phenotypes is summarized in Table 5. Overall, 172 patients (27.2%) experienced the primary composite outcome of all-cause readmission or mortality within 90 days post-discharge. A prominent risk gradient was observed across the trajectory groups (Figure 5). The incidence of the 90-day primary composite outcome was lowest in Phenotype 1 (14.9%), intermediate in Phenotype 2 (33.2%), and highest in Phenotype 3 (61.8%) (*p* < 0.001).

**Table 5:** Clinical outcomes across inflammatory-nutritional-renal trajectory phenotypes.

Clinical Outcomes	Total (*N* = 633)	Phenotype 1 (*n* = 348)	Phenotype 2 (*n* = 196)	Phenotype 3 (*n* = 89)	*P*-value
Primary outcome, *n* (%)					
90-day all-cause readmission or mortality	172 (27.2)	52 (14.9)	65 (33.2)	55 (61.8)	<0.001
90-day HF-related readmission or all-cause mortality, *n* (%)	149 (23.5)	42 (12.1)	55 (28.1)	52 (58.4)	<0.001
Secondary post-discharge outcomes, *n* (%)					
90-day HF-related readmission	110 (17.4)	32 (9.2)	42 (21.4)	36 (40.4)	<0.001
90-day all-cause mortality	45 (7.1)	10 (2.9)	17 (8.7)	18 (20.2)	<0.001
90-day cardiovascular mortality	32 (5.1)	6 (1.7)	12 (6.1)	14 (15.7)	<0.001
30-day all-cause readmission or mortality	75 (11.8)	20 (5.7)	28 (14.3)	27 (30.3)	<0.001
180-day all-cause readmission or mortality	240 (37.9)	95 (27.3)	85 (43.4)	60 (67.4)	<0.001
Secondary in-hospital outcomes, *n* (%)					
ICU/CCU admission or transfer	114 (18.0)	42 (12.1)	39 (19.9)	33 (37.1)	<0.001
Prolonged length of stay	158 (25.0)	52 (14.9)	59 (30.1)	47 (52.8)	<0.001
In-hospital WRF	130 (20.5)	35 (10.1)	49 (25.0)	46 (51.7)	<0.001
Inadequate pre-discharge NT-proBNP reduction	185 (29.2)	62 (17.8)	68 (34.7)	55 (61.8)	<0.001

Data are presented as *n* (%). Prolonged length of stay was defined as a hospitalization duration exceeding the 75th percentile of the study cohort. In-hospital WRF was defined based on KDIGO criteria as an increase in serum creatinine of ≥0.3 mg/dL (≥26.5 μmol/L) within 48 h or an increase to ≥1.5 times baseline within 7 days. HF-related readmissions were retrospectively adjudicated using the principal reason for hospitalization, objective evidence of worsening heart failure, and documented intensification of acute heart failure treatment. HF, heart failure; ICU, intensive care unit; CCU, coronary care unit; WRF, worsening renal function; NT-proBNP, N-terminal pro-B-type natriuretic peptide; KDIGO, Kidney Disease: Improving Global Outcomes.

When the readmission component was restricted to adjudicated HF-related readmission, 149 patients (23.5%) experienced HF-related readmission or all-cause mortality within 90 days. The incidence of this key disease-specific composite outcome increased progressively across Phenotype 1, Phenotype 2, and Phenotype 3 (12.1% [42/348], 28.1% [55/196], and 58.4% [52/89], respectively; *p* < 0.001). This gradient was comparable to that observed for the primary all-cause composite outcome.

Similar significant risk gradients were consistently identified across the prespecified secondary outcomes. For post-discharge endpoints, patients in Phenotype 3 exhibited the highest rates of 90-day HF-related readmission (40.4%), 90-day all-cause mortality (20.2%), 30-day all-cause readmission or mortality (30.3%), and 180-day all-cause readmission or mortality (67.4%), with all comparisons across the three phenotypes yielding *p* < 0.001. Regarding in-hospital secondary endpoints, Phenotype 3 had the highest incidence of ICU/CCU admission or transfer during the index hospitalization (37.1% vs. 19.9% in Phenotype 2 and 12.1% in Phenotype 1; *p* < 0.001). Furthermore, the proportion of patients experiencing a prolonged length of stay (>75th percentile of the cohort) and in-hospital WRF progressively increased from Phenotype 1 to Phenotype 3 (both *p* < 0.001). Inadequate pre-discharge reduction of NT-proBNP was also disproportionately prevalent in Phenotype 3 compared to the other two groups (*p* < 0.001).

### 3.5 Association between trajectory phenotypes and 90-day readmission or mortality

Associations between trajectory phenotypes and the 90-day composite outcome were evaluated using multivariable logistic and Cox regression models (Table 6). With Phenotype 1 as the reference, unadjusted logistic regression (Model 1) revealed significantly elevated risks for Phenotype 2 (OR, 2.82; 95% CI: 1.87–4.27, *p* < 0.001) and Phenotype 3 (OR, 9.21; 95% CI: 5.56–15.24, *p* < 0.001).

**Table 6:** Association between trajectory phenotypes and 90-day readmission or mortality.

Analytical models and phenotypes	Events/total, *n*/*N*	Unadjusted model 1		Adjusted model 2		Adjusted model 3	
		OR (95% CI)	*P*-value	OR (95% CI)	*P*-value	OR (95% CI)	*P*-value
Logistic regression							
Phenotype 1 (Reference)	52/348	1.00 (Reference)	—	1.00 (Reference)	—	1.00 (Reference)	—
Phenotype 2	65/196	2.82 (1.87–4.27)	<0.001	2.45 (1.59–3.78)	<0.001	2.12 (1.34–3.35)	0.001
Phenotype 3	55/89	9.21 (5.56–15.24)	<0.001	6.45 (3.75–11.08)	<0.001	4.85 (2.58–9.12)	<0.001
		HR (95% CI)	*P*-value	HR (95% CI)	*P*-value	HR (95% CI)	*P*-value
Cox proportional hazards							
Phenotype 1 (Reference)	52/348	1.00 (Reference)	—	1.00 (Reference)	—	1.00 (Reference)	—
Phenotype 2	65/196	2.35 (1.62–3.40)	<0.001	2.15 (1.45–3.18)	<0.001	1.88 (1.25–2.82)	0.002
Phenotype 3	55/89	5.42 (3.68–7.98)	<0.001	4.35 (2.88–6.56)	<0.001	3.56 (2.24–5.65)	<0.001

Model 1 was unadjusted. Model 2 adjusted for age, sex, BMI, prior HF hospitalization, LVEF phenotype, and NYHA functional class. Model 3 further adjusted for diabetes mellitus, coronary artery disease, atrial fibrillation, chronic kidney disease, admission SBP, log-transformed NT-proBNP, serum sodium, and discharge medications (SGLT2i, ARNI, ACEI/ARB, beta-blockers, MRA). OR, odds ratio; HR, hazard ratio; CI, confidence interval; BMI, body mass index; HF, heart failure; LVEF, left ventricular ejection fraction; NYHA, New York Heart Association; SBP, systolic blood pressure; NT-proBNP, N-terminal pro-B-type natriuretic peptide; SGLT2i, sodium-glucose cotransporter 2 inhibitor; ARNI, angiotensin receptor-neprilysin inhibitor; ACEI, angiotensin-converting enzyme inhibitor; ARB, angiotensin II receptor blocker; MRA, mineralocorticoid receptor antagonist.

This risk gradient remained after sequential adjustment. In the fully adjusted Model 3, which accounted for demographic characteristics, HF severity, comorbidities, admission systolic blood pressure, baseline NT-proBNP, serum sodium, and discharge medications, the adjusted ORs were 2.12 (95% CI, 1.34–3.35; *p* = 0.001) for Phenotype 2 and 4.85 (95% CI, 2.58–9.12; *p* < 0.001) for Phenotype 3. When baseline log-transformed NLR, serum albumin, and eGFR were additionally included, the associations were attenuated but remained statistically significant for Phenotype 2 (OR, 1.68; 95% CI, 1.02–2.76; *p* = 0.041) and Phenotype 3 (OR, 3.01; 95% CI, 1.46–6.21; *p* = 0.003) compared with Phenotype 1 (Supplementary Table S3, SA 11).

Time-to-event analyses via Cox proportional hazards regression yielded consistent findings. In the fully adjusted Cox model, Phenotypes 2 and 3 remained significantly associated with increased composite outcome hazards (HR, 1.88; 95% CI: 1.25–2.82, *p* = 0.002; and HR, 3.56; 95% CI: 2.24–5.65, *p* < 0.001, respectively) compared with Phenotype 1.

### 3.6 Incremental prognostic value of trajectory phenotypes

**Figure 6:**
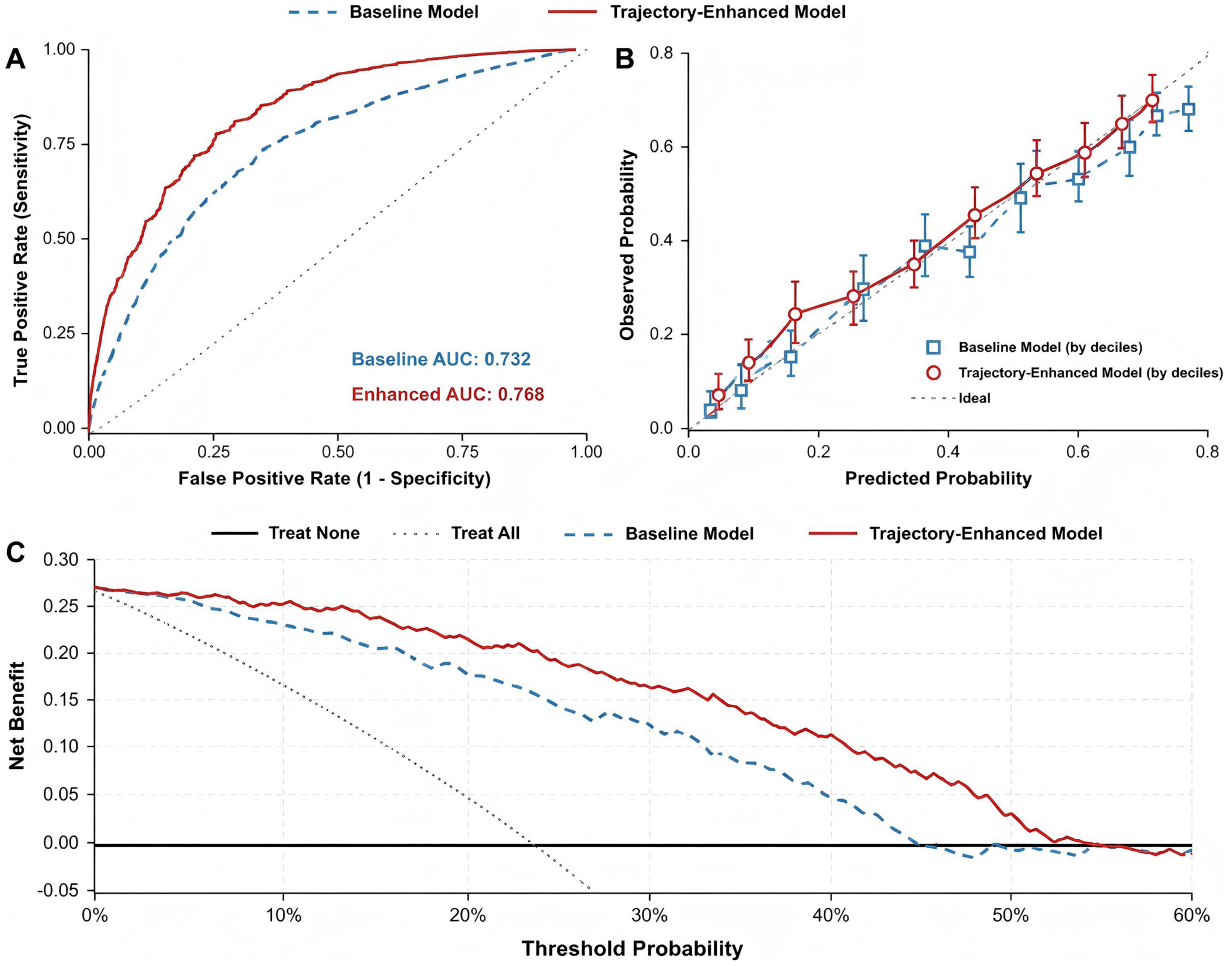
Incremental prognostic value of baseline biomarkers and trajectory phenotype. Multi-panel figure comparing the clinical model, the clinical-plus-baseline-biomarker model, and the trajectory-enhanced model for the 90-day composite outcome. **(A)** Receiver operating characteristic curves comparing model discrimination. **(B)** Calibration plots showing agreement between predicted and observed risks. **(C)** Decision curve analysis comparing net clinical benefit across threshold probabilities. The clinical model included age, sex, LVEF phenotype, NYHA functional class, prior HF hospitalization, chronic kidney disease, admission systolic blood pressure, and baseline log-transformed NT-proBNP. The clinical-plus-baseline-biomarker model additionally included baseline log-transformed NLR, serum albumin, and eGFR. The trajectory-enhanced model further included trajectory phenotype. LVEF, left ventricular ejection fraction; NYHA, New York Heart Association; HF, heart failure; NT-proBNP, N-terminal pro-B-type natriuretic peptide; NLR, neutrophil-to-lymphocyte ratio; eGFR, estimated glomerular filtration rate.

The incremental prognostic value of the trajectory phenotypes was evaluated using three nested models (Table 7 and Figure 6). The clinical model had an AUC of 0.732 (95% CI, 0.688–0.776) and a Brier score of 0.165. Adding baseline log-transformed NLR, serum albumin, and eGFR increased the AUC to 0.754 (95% CI, 0.711–0.797; *p* = 0.041 vs. the clinical model) and reduced the Brier score to 0.157. Further addition of the trajectory phenotype increased the AUC to 0.768 (95% CI, 0.725–0.811; *p* = 0.036 vs. the clinical-plus-baseline-biomarker model) and reduced the Brier score to 0.152. The likelihood-ratio test also supported improved model fit after addition of the trajectory phenotype to the clinical-plus-baseline-biomarker model (*χ*^2^ = 8.01; *p* = 0.018).

**Table 7:** Incremental prognostic value of baseline biomarkers and trajectory phenotype for the 90-day composite outcome.

Model	Included predictors	AUC (95% CI)	*P*-value for ΔAUC	Brier score	Likelihood-ratio test
Clinical model	Age, sex, LVEF phenotype, NYHA functional class, prior HF hospitalization, chronic kidney disease, admission SBP, and baseline log-transformed NT-proBNP	0.732 (0.688–0.776)	Reference	0.165	Reference
Clinical + baseline biomarkers	Clinical model + baseline log-transformed NLR, serum albumin, and eGFR	0.754 (0.711–0.797)	0.041	0.157	*χ*^2^ = 9.16; *p* = 0.027
Clinical + baseline biomarkers + trajectory phenotype	Clinical + baseline biomarker model + trajectory phenotype	0.768 (0.725–0.811)	0.036	0.152	*χ*^2^ = 8.01; *p* = 0.018

The primary outcome was all-cause readmission or all-cause mortality within 90 days after discharge. *P*-values for changes in AUC were calculated using DeLong's test and compare each model with the immediately preceding nested model. Likelihood-ratio tests also compare each model with the immediately preceding model. A lower Brier score indicates lower overall prediction error. AUC, area under the receiver operating characteristic curve; CI, confidence interval; LVEF, left ventricular ejection fraction; NYHA, New York Heart Association; HF, heart failure; SBP, systolic blood pressure; NT-proBNP, N-terminal pro-B-type natriuretic peptide; NLR, neutrophil-to-lymphocyte ratio; eGFR, estimated glomerular filtration rate.

Calibration plots showed acceptable agreement between predicted and observed risks for all three models, with the trajectory-enhanced model demonstrating the lowest prediction error. In decision curve analysis, the trajectory-enhanced model provided a modestly greater net benefit than the clinical-plus-baseline-biomarker model across threshold probabilities of approximately 15%–45% (Figure 6).

### 3.7 Sensitivity analyses

The robustness of the primary multivariable findings was evaluated through prespecified sensitivity analyses (Supplementary Table S3). The risk estimates were not materially altered in complete-case or multiply imputed analyses, after excluding patients with baseline eGFR <30 mL/min/1.73 m^2^, after replacing the pre-discharge T3 node with a fixed 96–168-hour post-admission window, or after substituting serum creatinine for eGFR in the trajectory model. When all-cause readmission was replaced by adjudicated HF-related readmission, 149 patients (23.5%) experienced the disease-specific composite outcome. Phenotype 2 (OR, 2.00; 95% CI, 1.24–3.22; *p* = 0.004) and Phenotype 3 (OR, 4.62; 95% CI, 2.41–8.87; *p* < 0.001) remained associated with 90-day HF-related readmission or all-cause mortality compared with Phenotype 1 (Supplementary Table S3, SA 3).

The inverse probability-of-observation weighted analysis included all 822 clinically eligible discharge survivors with ascertainable outcomes. After weighting, all absolute standardized mean differences for the modeled predictors of complete trajectory availability were <0.10. The stabilized weights had a mean of 1.00 and a 1st–99th percentile range of 0.71–1.83. The weighted associations remained significant for Phenotype 2 (OR, 2.06; 95% CI, 1.28–3.31; *p* = 0.003) and Phenotype 3 (OR, 4.51; 95% CI, 2.34–8.69; *p* < 0.001) (Supplementary Table S3, SA 7).

The exploratory T1–T2-only analysis included 742 patients with complete biomarker measurements through 72 h. Three independently estimated early classes were identified: Class 1, 410 patients (55.3%); Class 2, 231 patients (31.1%); and Class 3, 101 patients (13.6%). The 90-day composite outcome occurred in 72 patients (17.6%), 71 patients (30.7%), and 53 patients (52.5%), respectively. After full baseline adjustment, Class 2 (OR, 1.83; 95% CI, 1.17–2.86; *p* = 0.008) and Class 3 (OR, 3.74; 95% CI, 2.12–6.61; *p* < 0.001) remained associated with the outcome compared with Class 1 (Supplementary Table S3, SA 8).

Additional adjustment for in-hospital treatment intensity, ICU/CCU care, and NT-proBNP response attenuated but did not eliminate the associations for Phenotype 2 (OR, 1.89; 95% CI, 1.17–3.06; *p* = 0.009) and Phenotype 3 (OR, 3.96; 95% CI, 2.02–7.76; *p* < 0.001) (Supplementary Table S3, SA 9). Additional adjustment for SGLT2 inhibitor, GLP-1RA, and systemic corticosteroid exposure before T3 produced minimal changes in the estimates for Phenotype 2 (OR, 2.07; 95% CI, 1.30–3.31; *p* = 0.002) and Phenotype 3 (OR, 4.63; 95% CI, 2.43–8.83; *p* < 0.001) (Supplementary Table S3, SA 10). When baseline log-transformed NLR, serum albumin, and eGFR were additionally included in Model 3, the associations were attenuated but remained significant for Phenotype 2 (OR, 1.68; 95% CI, 1.02–2.76; *p* = 0.041) and Phenotype 3 (OR, 3.01; 95% CI, 1.46–6.21; *p* = 0.003) (Supplementary Table S3, SA 11).

## 4 Discussion

In this retrospective cohort of patients hospitalized with ADHF who survived to discharge, three in-hospital inflammatory-nutritional-renal trajectory phenotypes were identified using serial NLR, serum albumin, and eGFR measurements. The phenotypes showed a graded association with the 90-day composite outcome, increasing from 14.9% in Phenotype 1 to 33.2% in Phenotype 2 and 61.8% in Phenotype 3. The overall 90-day event rate of 27.2% was consistent with the recognized early vulnerability after acute HF hospitalization. Espersen et al. reported that approximately one quarter of patients with acute HF experienced HF hospitalization or all-cause death within 90 days, while Khan et al. reported a 90-day readmission rate of 31.2% in a large national cohort (4, 5). These findings support the clinical relevance of risk assessment extending through the early post-discharge period.

The identified phenotypes should not be interpreted as wholly novel biological subtypes independent of baseline disease severity. Phenotype 3 included older patients with greater comorbidity burden, more advanced HF, lower blood pressure, worse renal function, higher NT-proBNP concentrations, and a greater prevalence of HFrEF. This clustering is clinically plausible because outcomes after decompensation are jointly influenced by congestion, comorbidities, renal dysfunction, neurohormonal activation, and the adequacy of stabilization before discharge (11, 12). Accordingly, the phenotypes are more appropriately regarded as dynamic risk phenotypes integrating baseline vulnerability with the subsequent in-hospital evolution of inflammatory, nutritional, and renal markers. Additional adjustment for baseline NLR, serum albumin, and eGFR attenuated the associations, indicating that admission severity explained part of the observed risk gradient. However, Phenotypes 2 and 3 remained associated with the primary outcome, suggesting that the longitudinal patterns contained information not fully captured by the corresponding admission measurements.

The inflammatory component of the phenotypes is consistent with previous evidence linking NLR with HF prognosis. Higher NLR has been associated with increased mortality in HF and with adverse short- and long-term outcomes in ADHF (7, 20). In the present cohort, patients who subsequently experienced readmission or mortality had persistently higher NLR levels and a less pronounced decline during hospitalization. This pattern may reflect sustained inflammatory activation or incomplete biological stabilization, although NLR remains nonspecific and may also be influenced by infection, physiological stress, and medication exposure. Similar evidence has been reported for longitudinal inflammatory profiles, with persistently elevated hsCRP trajectories associated with higher mortality in acute HF (21).

Serum albumin provided a complementary marker of nutritional-inflammatory reserve. Malnutrition assessed using the CONUT score has been associated with mortality in acute HF, and albumin-based indices have demonstrated prognostic value across HF populations (8, 22). Hypoalbuminaemia is common in HF but may reflect several overlapping mechanisms, including inflammation, hepatic congestion, capillary leakage, renal loss, hemodilution, frailty, and inadequate nutritional reserve (23). Therefore, the lower and declining albumin pattern observed in Phenotype 3 should be interpreted as a marker of systemic vulnerability rather than evidence that albumin replacement itself would improve outcomes.

The renal component also requires contextual interpretation. Phenotype 3 had lower baseline eGFR, greater CKD burden, higher serum creatinine and BUN, and more frequent in-hospital worsening renal function. Renal changes during ADHF may arise from venous congestion, hypoperfusion, neurohormonal activation, diuretic exposure, or intrinsic kidney injury, and should therefore be interpreted within the broader treatment and congestion trajectory (9). Previous studies have demonstrated heterogeneous renal function trajectories in acute HF and have questioned whether renal trajectories alone consistently retain prognostic value after adjustment (24). The present approach differs by jointly integrating renal function with inflammatory and nutritional markers, but it cannot fully distinguish intrinsic biological vulnerability from the effects of disease severity and treatment.

The outcome gradient was consistent across HF-related readmission, mortality, 30-day and 180-day events, prolonged hospitalization, ICU or CCU care, worsening renal function, and inadequate NT-proBNP reduction. Restricting the readmission component to adjudicated HF-related hospitalization produced similar associations, suggesting that the primary results were not driven solely by non-cardiovascular readmissions. The less favorable NT-proBNP response observed in the high-risk phenotype was also biologically coherent with evidence that natriuretic peptide reduction during hospitalization is associated with prognosis in acute HF (6, 25). Nevertheless, because treatment intensity, biomarker evolution, and clinical recovery occurred concurrently, these parallel associations cannot establish a causal pathway.

The incremental prognostic findings should be interpreted conservatively. The clinical model had an AUC of 0.732, which increased to 0.754 after adding baseline NLR, serum albumin, and eGFR and to 0.768 after further addition of the trajectory phenotype. Thus, the incremental gain attributable specifically to the trajectory phenotype beyond the corresponding baseline biomarkers was 0.014. The accompanying reductions in Brier score and modest improvement in decision-curve net benefit support measurable additional information, but the magnitude is not sufficient to establish clear clinical superiority over simpler approaches. Data-driven phenotyping has identified clinically relevant HF subgroups in other settings, including cardiac intensive care populations and longitudinal transitions in HFpEF (10, 26). However, the present analysis was not designed as formal prediction-model development and does not establish that LCMM-based classification should replace conventional clinical assessment, natriuretic peptide evaluation, or simpler risk scores.

Practical implementation would require automation rather than manual model fitting by clinicians. NLR, serum albumin, and creatinine-derived eGFR are routinely measured and impose little additional laboratory cost in many hospitals. After external validation, a fixed classification algorithm could potentially be embedded within the electronic medical record to retrieve prespecified T1, T2, and T3 values and generate a phenotype or risk category before discharge. The principal implementation barriers would be standardization of sampling windows, handling of incomplete measurements, transportability of the class definitions across institutions, and integration into clinical workflows. A simpler approach based on admission or pre-discharge biomarker thresholds would be easier to implement and may offer comparable utility in some settings. Therefore, future studies should directly compare the trajectory strategy with established HF risk scores, single-time-point biomarkers, and simpler change-score approaches, and should assess whether phenotype-informed follow-up changes clinical decisions or outcomes.

The prognostic relevance of the phenotypes may also vary across HFrEF, HFmrEF, and HFpEF. Although LVEF phenotype was included in the adjusted models, Phenotype 3 contained a higher proportion of patients with HFrEF, and the present sample was not designed or powered to estimate phenotype-specific trajectory effects within each LVEF subgroup. Inflammatory burden, renal vulnerability, congestion patterns, treatment response, and comorbidity profiles differ across the HF spectrum, which may affect both trajectory membership and prognostic performance. Consequently, the observed associations should not be assumed to be identical across HFrEF, HFmrEF, and HFpEF. Larger multicenter cohorts should formally evaluate interaction by LVEF phenotype and determine whether separate trajectory definitions or risk thresholds are required.

The sensitivity analyses supported the directional robustness of the findings across alternative missing-data approaches, outcome definitions, renal specifications, timing of the third measurement, treatment-intensity adjustment, medication adjustment, and analyses addressing complete trajectory availability. The three-class structure was also reselected in 87.6% of bootstrap samples, indicating satisfactory internal stability. However, bootstrap validation assesses reproducibility within the same source population and does not demonstrate transportability. Differences in patient case mix, admission thresholds, treatment pathways, laboratory schedules, and discharge practices may alter the number, prevalence, and morphology of the classes in other settings.

Several limitations require emphasis. First, the single-center retrospective design precludes causal inference and limits generalizability. The findings demonstrate associations between in-hospital biomarker trajectories and subsequent outcomes but cannot establish that the trajectories themselves cause adverse events or that modifying them would improve prognosis. Second, no external validation was performed. Internal bootstrap stability and sensitivity analyses do not substitute for validation in an independent population, and the proposed class structure should not be used for clinical decision-making until its reproducibility and calibration have been confirmed elsewhere. Third, the primary cohort included only patients who survived to discharge and had complete NLR, albumin, and eGFR measurements at all three nodes. This requirement may have preferentially selected patients with longer stays, more intensive monitoring, or specific clinical pathways and excluded both rapidly improving patients and those with early deterioration. Although inverse probability-of-observation weighting produced similar estimates after balancing measured predictors of complete data, residual selection related to unmeasured testing decisions, discharge timing, and clinical instability remains possible. Fourth, T2 and T3 measurements occurred after treatment initiation, so trajectory membership reflected a mixture of baseline disease burden, treatment response, and evolving clinical severity. Adjustment for treatment intensity, NT-proBNP response, and relevant medication exposure reduced but could not eliminate time-dependent and residual confounding. Fifth, readmissions occurring outside the institutional system may have been missed, and retrospective adjudication of HF-related readmission remained susceptible to incomplete documentation and misclassification. Sixth, NLR and serum albumin are nonspecific and may be affected by occult infection, inflammation, corticosteroid exposure, congestion, hemodilution, hepatic dysfunction, renal loss, and nutritional status. Finally, LCMM classification depends on the selected variables, number and timing of measurements, scaling, covariance assumptions, and class-selection criteria. Prospective multicenter studies are needed to externally validate the phenotypes, assess performance across LVEF subgroups, compare them with simpler risk-stratification methods, and determine whether trajectory-informed discharge planning improves patient outcomes.

## 5 Conclusion

In patients hospitalized with ADHF who survived to discharge, serial inflammatory, nutritional, and renal measurements were used to identify three in-hospital trajectory phenotypes associated with graded risks of 90-day readmission or mortality. Baseline disease severity explained part of these associations, while the longitudinal phenotype provided limited additional prognostic information beyond conventional clinical factors and baseline biomarker levels. These findings are hypothesis-generating and require prospective external validation before the trajectory phenotypes can be considered for routine clinical risk stratification.

## Data Availability

The raw data supporting the conclusions of this article will be made available by the authors, without undue reservation.

## References

[1] Shahim B Kapelios CJ Savarese G Lund LH. Global public health burden of heart failure: an updated review. Card Fail Rev. (2023) 9:e11. 10.15420/cfr.2023.05 37547123 PMC10398425

[2] Fletcher RA Rockenschaub P Neuen BL Walter IJ Conrad N Mizani MA. Contemporary epidemiology of hospitalised heart failure with reduced versus preserved ejection fraction in England: a retrospective, cohort study of whole-population electronic health records. Lancet Public Health. (2024) 9(11):e871–85. 10.1016/S2468-2667(24)00215-9 39486903

[3] Lan T Liao Y-H Zhang J Yang Z-P Xu G-S Zhu L. Mortality and readmission rates after heart failure: a systematic review and meta-analysis. Ther Clin Risk Manag. (2021) 17:1307–20. 10.2147/TCRM.S340587 34908840 PMC8665875

[4] Khan MS Sreenivasan J Lateef N Abougergi MS Greene SJ Ahmad T. Trends in 30- and 90-day readmission rates for heart failure. Circ Heart Fail. (2021) 14(4):e008335. 10.1161/CIRCHEARTFAILURE.121.008335 33866827

[5] Espersen C Campbell RT Claggett BL Lewis EF Docherty KF Lee MMY. Predictors of heart failure readmission and all-cause mortality in patients with acute heart failure. Int J Cardiol. (2024) 406:132036. 10.1016/j.ijcard.2024.132036 38599465 PMC11146586

[6] Wettersten N. Biomarkers in acute heart failure: diagnosis, prognosis, and treatment. Int J Heart Fail. (2021) 3(2):81–105. 10.36628/ijhf.2020.0036 36262882 PMC9536694

[7] Vakhshoori M Nemati S Sabouhi S Yavari B Shakarami M Bondariyan N. Neutrophil to lymphocyte ratio prognostic effects on heart failure: a systematic review and meta-analysis. BMC Cardiovasc Disord. (2023) 23(1):555. 10.1186/s12872-023-03572-6 37957565 PMC10644447

[8] Jiang J Miao P Xin G. Prognostic value of albumin-based indices for mortality after heart failure: a systematic review and meta-analysis. BMC Cardiovasc Disord. (2024) 24(1):570. 10.1186/s12872-024-04244-9 39420315 PMC11484320

[9] Mullens W Damman K Testani JM Martens P Mueller C Lassus J. Evaluation of kidney function throughout the heart failure trajectory: a position statement from the heart failure association of the European Society of Cardiology. Eur J Heart Fail. (2020) 22(4):584–603. 10.1002/ejhf.1697 31908120

[10] Jentzer JC Reddy YNV Soussi S Crespo-Diaz R Patel PC Lawler PR. Unsupervised machine learning to identify subphenotypes among cardiac intensive care unit patients with heart failure. ESC Heart Fail. (2024) 11(6):4242–56. 10.1002/ehf2.15027 39160644 PMC11631334

[11] McDonagh TA Metra M Adamo M Gardner RS Baumbach A Böhm M. 2021 ESC guidelines for the diagnosis and treatment of acute and chronic heart failure. Eur Heart J. (2021) 42(36):3599–726. 10.1093/eurheartj/ehab368 34447992

[12] Heidenreich PA Bozkurt B Aguilar D Allen LA Byun JJ Colvin MM. 2022 AHA/ACC/HFSA guideline for the management of heart failure: a report of the American College of Cardiology/American Heart Association Joint Committee on clinical practice guidelines. J Am Coll Cardiol. (2022) 79(17):e263–421. 10.1016/j.jacc.2021.12.012 35379503

[13] Bozkurt B Coats AJS Tsutsui H Abdelhamid M Adamopoulos S Albert N. Universal definition and classification of heart failure: a report of the heart failure society of America, heart failure association of the European Society of Cardiology, Japanese heart failure society and writing committee of the universal definition of heart failure. J Card Fail. (2021) 27(4):387–413. 10.1016/j.cardfail.2021.01.022 33663906

[14] Charlson ME Pompei P Ales KL MacKenzie CR. A new method of classifying prognostic comorbidity in longitudinal studies: development and validation. J Chronic Dis. (1987) 40(5):373–83. 10.1016/0021-9681(87)90171-8 3558716

[15] Inker LA Eneanya ND Coresh J Tighiouart H Wang D Sang Y. New creatinine- and cystatin C-based equations to estimate GFR without race. N Engl J Med. (2021) 385(19):1737–49. 10.1056/NEJMoa2102953 34554658 PMC8822996

[16] Proust-Lima C Philipps V Liquet B. Estimation of extended mixed models using latent classes and latent processes: the R package lcmm. J Stat Softw. (2017) 78(2):1–56. 10.18637/jss.v078.i02

[17] Nagin DS Odgers CL. Group-based trajectory modeling in clinical research. Annu Rev Clin Psychol. (2010) 6:109–38. 10.1146/annurev.clinpsy.121208.131413 20192788

[18] Khwaja A. KDIGO Clinical practice guidelines for acute kidney injury. Nephron Clin Pract. (2012) 120(4):c179–84. 10.1159/000339789 22890468

[19] Nguena Nguefack HL Pagé MG Katz J Choinière M Vanasse A Dorais M. Trajectory modelling techniques useful to epidemiological research: a comparative narrative review of approaches. Clin Epidemiol. (2020) 12:1205–22. 10.2147/CLEP.S265287 33154677 PMC7608582

[20] Ang SP Chia JE Jaiswal V Hanif M Iglesias J. Prognostic value of neutrophil-to-lymphocyte ratio in patients with acute decompensated heart failure: a meta-analysis. J Clin Med. (2024) 13(5):1212. 10.3390/jcm13051212 38592030 PMC10931846

[21] He G Ji R Huo X Su X Ge J Li W. Long-term trajectories of high-sensitivity C-reactive protein level among patients with acute heart failure. J Inflamm Res. (2023) 16:359–71. 10.2147/JIR.S387534 36741288 PMC9891160

[22] Iwakami N Nagai T Furukawa TA Sugano Y Honda S Okada A. Prognostic value of malnutrition assessed by controlling nutritional Status score for long-term mortality in patients with acute heart failure. Int J Cardiol. (2017) 230:529–36. 10.1016/j.ijcard.2016.12.064 28041709

[23] Biancucci M Barbiero R Pennella B Cannatà A Ageno W Tangianu F. Hypoalbuminaemia and heart failure: a practical review of current evidence. Eur J Heart Fail. (2025) 27(2):293–306. 10.1002/ejhf.3363 38962822 PMC11860734

[24] Beldhuis IE Streng KW Van Der Meer P Ter Maaten JM O'Connor CM Metra M. Trajectories of changes in renal function in patients with acute heart failure. J Card Fail. (2019) 25(11):866–74. 10.1016/j.cardfail.2019.07.004 31319165

[25] Kagiyama N Kitai T Hayashida A Yamaguchi T Okumura T Kida K. Prognostic value of BNP reduction during hospitalization in patients with acute heart failure. J Card Fail. (2019) 25(9):712–21. 10.1016/j.cardfail.2019.04.004 30965102

[26] Matsuoka Y Sotomi Y Nakatani D Okada K Sunaga A Kida H. Phenotypic trajectories from acute to stable phase in heart failure with preserved ejection fraction: insights from the PURSUIT-HFpEF study. J Am Heart Assoc. 2025;14(3):e037567. 10.1161/JAHA.124.037567 39895530 PMC12074776

