# Supplementary material for: In-hospital inflammatory-nutritional-renal trajectory phenotypes and 90-day readmission or mortality in patients with acute decompensated heart failure: a retrospective cohort study
Source: Front Cardiovasc Med. 2026 Jul 31;13:1900715. doi: 10.3389/fcvm.2026.1900715 (PMC13473160; doi:10.3389/fcvm.2026.1900715)
Supplement: Supplementary file 1 [file Table1.docx]

# Supplementary Material

## S1. Electronic data sources and case ascertainment

Clinical data were retrieved from the institutional hospital information system, including the electronic medical record, laboratory information system, echocardiography database, computerized physician order entry system, medication administration records, nursing flowsheets, discharge summaries, outpatient records, post-discharge follow-up records, and institutional readmission registry. Records from these systems were linked using the unique hospital identification number assigned to each patient.

The initial cohort was identified using International Classification of Diseases, Tenth Revision heart failure codes, primarily I50.x, supplemented by institution-specific heart-failure-related discharge diagnostic terms and code mappings. These codes were used only for broad case screening and were not considered sufficient to confirm acute decompensated heart failure (ADHF). Duplicate records and admissions involving patients aged <18 years were removed before clinical review.

Two trained physicians independently reviewed the complete medical records of all potentially eligible admissions using a standardized eligibility form. The review included the admission history, physical examination, nursing documentation, chest imaging, electrocardiography, natriuretic peptide measurements, echocardiography, inpatient treatment records, and discharge summary. Eligible cases were required to represent new-onset or worsening heart failure that was the principal reason for hospitalization and required urgent inpatient treatment. Disagreements were resolved by joint review and consensus. Cases remaining uncertain after consensus review were adjudicated by a senior cardiologist who was not involved in the initial assessment.

ADHF was defined according to the 2021 European Society of Cardiology guideline, the 2022 American Heart Association/American College of Cardiology/Heart Failure Society of America guideline, and the Universal Definition and Classification of Heart Failure [1–3]. Confirmation required acute or worsening symptoms compatible with heart failure, such as dyspnea, orthopnea, reduced exercise tolerance, or peripheral edema, together with at least one documented sign or objective finding of pulmonary or systemic congestion. Eligible findings included pulmonary rales, elevated jugular venous pressure, peripheral edema, pulmonary vascular congestion or pulmonary edema on chest imaging, and pleural effusion. Evidence of an underlying cardiac structural or functional abnormality on echocardiography and corroborating evidence of heart failure through elevated BNP or NT-proBNP and/or objective congestion were also required. The clinical presentation had to necessitate urgent inpatient heart failure management, including intravenous loop diuretic, vasodilator, or other decongestive treatment.

For analyses addressing potential selection associated with complete serial biomarker measurements, the source cohort comprised 822 clinically eligible patients who survived to discharge and had ascertainable 90-day outcomes. Of these, 633 had complete NLR, serum albumin, and eGFR measurements at T1, T2, and T3 and were included in the primary trajectory cohort, whereas 189 lacked a complete three-node biomarker profile.

## S2. Detailed variable extraction and definitions

Baseline variables were defined as measurements or clinical information documented at admission or within the first 24 hours after admission unless otherwise specified. Demographic and clinical variables included age, sex, body mass index, admission source, smoking status, alcohol consumption, previous heart failure hospitalization, systolic and diastolic blood pressure, heart rate, respiratory rate, body temperature, peripheral oxygen saturation, New York Heart Association functional class, pulmonary rales, lower-extremity edema, jugular venous distension, hepatomegaly, pleural effusion, radiographic pulmonary edema, initial oxygen requirement, and emergency department or coronary care unit admission.

Comorbidities were identified from the documented medical history, discharge diagnoses, prior records, and medication history. These included hypertension, diabetes mellitus, coronary artery disease, previous myocardial infarction, atrial fibrillation, stroke, chronic kidney disease, chronic obstructive pulmonary disease, anemia, and peripheral vascular disease. Overall comorbidity burden was quantified using the Charlson Comorbidity Index [4].

Laboratory parameters included complete blood count, renal and hepatic function indices, serum electrolytes, inflammatory markers, cardiac biomarkers, and natriuretic peptides. Extracted variables included hemoglobin, red-cell distribution width, platelet count, absolute neutrophil count, absolute lymphocyte count, serum albumin, total protein, serum creatinine, blood urea nitrogen, uric acid, sodium, potassium, chloride, alanine aminotransferase, aspartate aminotransferase, total bilirubin, C-reactive protein, procalcitonin, D-dimer, cardiac troponin I or T, creatine kinase-MB, BNP, NT-proBNP, fasting glucose, glycated hemoglobin, total cholesterol, triglycerides, low-density lipoprotein cholesterol, and high-density lipoprotein cholesterol. BNP and NT-proBNP were retained as separate variables.

NLR was calculated by dividing the absolute neutrophil count by the absolute lymphocyte count. eGFR was extracted directly from the laboratory information system when available. When necessary, it was recalculated from serum creatinine using the 2021 Chronic Kidney Disease Epidemiology Collaboration creatinine equation without a race coefficient [5].

Echocardiographic variables were extracted from routine clinical reports and included left ventricular ejection fraction, left atrial diameter, left ventricular end-diastolic diameter, interventricular septal thickness, posterior wall thickness, E/e′ ratio, valvular regurgitation severity, and estimated pulmonary artery systolic pressure. Heart failure phenotype was classified according to left ventricular ejection fraction as HFrEF, HFmrEF, or HFpEF in accordance with contemporary guideline definitions [2].

In-hospital therapeutic variables included cumulative intravenous loop-diuretic dose converted to furosemide equivalents, intravenous vasodilator use, inotrope or vasopressor use, non-invasive ventilation, invasive mechanical ventilation, ultrafiltration, and ICU or coronary care unit care. Medication exposure before T3 included sodium-glucose cotransporter 2 inhibitors, glucagon-like peptide-1 receptor agonists, systemic corticosteroids, and other systemic immunosuppressive agents. Systemic corticosteroid exposure included oral or intravenous glucocorticoids but excluded inhaled corticosteroids. Discharge medications included sodium-glucose cotransporter 2 inhibitors, angiotensin receptor-neprilysin inhibitors, angiotensin-converting enzyme inhibitors or angiotensin receptor blockers, beta-blockers, mineralocorticoid receptor antagonists, digoxin, and oral anticoagulants.

## S3. Selection of biomarker measurements at the three clinical nodes

All trajectory windows were anchored to the exact date and time of hospital admission recorded in the electronic medical record rather than to treatment response, medication administration, transfer of care, or other patient-dependent clinical events.

T1 was defined as the first available measurement obtained within 0–24 hours after admission.

T2 was defined as a complete set of NLR, serum albumin, and serum creatinine-derived eGFR measurements obtained between 48.0 and 72.0 hours after admission. Sixty hours after admission was designated as the nominal center of this window. This interval was selected to capture the early biological response after approximately 2–3 days of routine decongestive and supportive treatment while maintaining a standardized time scale across patients.

T3 was defined as the last routine assessment obtained within 24–48 hours before discharge among patients who survived to discharge. For patients with shorter hospital stays who did not undergo repeat testing within this interval, the most recent pre-discharge measurement was used, provided that it occurred after T2 and no earlier than 72 hours after admission.

A standardized hierarchical algorithm was applied to identify the measurements used at each node. Measurements of NLR, serum albumin, and eGFR obtained from the same phlebotomy episode were preferred. When more than one complete panel was available during the T2 window, the panel collected closest to 60 hours after admission was selected. If the three components were not measured during the same phlebotomy episode, measurements obtained within a maximum interval of 6 hours were considered contemporaneous, and the set with the smallest combined temporal distance from 60 hours was retained. Routine morning measurements were prioritized only when two eligible panels were equally close to the nominal time point.

Values outside the prespecified T2 window were not carried forward, interpolated, or substituted. The same measurement-selection rules were applied before outcome status and trajectory-class assignment were examined. Patients without valid NLR, serum albumin, and eGFR measurements at all three nodes were excluded from the primary trajectory analysis.

The fixed 96–168-hour post-admission window was used only in sensitivity analysis as an alternative definition of T3.

## S4. Multivariate latent class mixed modelling

The core trajectory variables were NLR, serum albumin, and eGFR. NT-proBNP was not included in the primary trajectory model because serial sampling was less consistent across patients. It was instead used as a baseline adjustment covariate and as a treatment-response variable in sensitivity analyses.

Because of its right-skewed distribution, NLR was natural-log transformed. Log-transformed NLR, serum albumin, and eGFR were then standardized as Z-scores using the mean and standard deviation of the primary analytic cohort. Standardization allowed the three biomarkers to contribute on comparable scales.

Multivariate latent class mixed models were fitted using the lcmm package in R [6]. Candidate models specifying one, two, three, and four latent classes were fitted sequentially using identical longitudinal variables, time coding, and covariance structure. Each biomarker was modelled as a function of the prespecified clinical node. The same time specification was applied to all candidate models.

To reduce the probability of convergence to a local maximum, each candidate model was estimated using 100 sets of randomized initial values derived from the converged one-class model. The solution with the highest replicated log-likelihood was retained when convergence criteria for parameter estimates, the log-likelihood, and the Hessian matrix were satisfied.

The optimal number of classes was selected using a prespecified hierarchical framework. Bayesian information criterion was the primary statistical criterion. Akaike information criterion and sample-size-adjusted Bayesian information criterion were considered supportive. Candidate solutions were additionally required to demonstrate adequate class separation, defined by entropy and mean posterior class-membership probabilities ≥0.70, a minimum class size of ≥5% of the analytic cohort, clinically distinguishable trajectory patterns, and reproducibility across randomized starting values [7].

A more complex solution was not selected solely because it had a lower Akaike information criterion if it produced a class containing <5% of patients, showed poorer class separation, failed to improve Bayesian information criterion, or lacked a clinically distinguishable trajectory pattern. Each patient was assigned to the class with the highest posterior membership probability.

Class labels in subsequent analyses were based on the relative temporal patterns of log-transformed NLR, serum albumin, and eGFR. Clinical characteristics and outcomes were not used to determine the number of classes or to assign individual patients.

## S5. Outcome ascertainment and adjudication

The primary outcome was the first occurrence of all-cause readmission or all-cause mortality within 90 days after discharge. Discharge was defined as time zero. An all-cause readmission was any unplanned inpatient hospitalization occurring within the follow-up period. Planned admissions for scheduled procedures, staged interventions, rehabilitation, or routine evaluation were excluded.

Outcome information was obtained from institutional readmission records, outpatient records, standardized telephone follow-up, subsequent hospitalization records, and institutional death registry data when available. All-cause mortality was recorded regardless of the documented cause.

The key disease-specific secondary outcome was HF-related readmission or all-cause mortality within 90 days. HF-related readmission was defined as an unplanned hospitalization in which worsening symptoms or signs of heart failure were the principal reason for admission and required intensification of HF treatment. Qualifying treatment included intravenous diuretics, intravenous vasodilators, inotropic therapy, ultrafiltration, or other acute decongestive management.

Potential HF-related readmissions captured within the institutional system were independently reviewed by two physicians using the admission history, physical examination, natriuretic peptide results, chest imaging, treatment records, and discharge diagnosis. Disagreements were resolved by consensus, with adjudication by a senior cardiologist when necessary.

Prolonged length of stay was defined as a hospitalization duration above the 75th percentile of the study cohort. In-hospital worsening renal function was defined using the serum creatinine component of the Kidney Disease: Improving Global Outcomes acute kidney injury criteria as an increase in serum creatinine of ≥0.3 mg/dL, equivalent to ≥26.5 μmol/L, within 48 hours or an increase to ≥1.5 times the baseline value within 7 days [8].

## S6. Missing-data procedures

Patients with missing primary outcome data or without complete NLR, serum albumin, and eGFR measurements at T1, T2, and T3 were excluded from the primary trajectory analysis. Variables with >20% missingness were not entered into the main multivariable models.

For candidate covariates with 5%–20% missingness, multiple imputation by chained equations was performed using 20 imputed datasets. The imputation model included the primary composite outcome, trajectory phenotype, demographic variables, major comorbidities, echocardiographic parameters, core baseline laboratory variables, and covariates used in the multivariable outcome models. Highly skewed variables, including NT-proBNP, were natural-log transformed before imputation.

Regression coefficients and standard errors from the imputed datasets were pooled using Rubin’s rules. When all variables used in a particular model had <5% missingness, complete-case analysis was applied. Complete-case and multiply imputed analyses were also reported separately as sensitivity analyses.

## S7. Bootstrap assessment of trajectory stability

Internal stability of the selected latent-class solution was evaluated using 500 nonparametric bootstrap samples generated by resampling patients with replacement.

In each bootstrap sample, the complete trajectory-modelling procedure was repeated. This included fitting the one- to four-class candidate models, applying the prespecified class-selection criteria, selecting the optimal number of classes, and estimating posterior class membership. Thus, the bootstrap assessment incorporated uncertainty arising from both class-number selection and patient classification.

For bootstrap samples in which a three-class solution was selected, the derived class labels were aligned with the original solution by minimizing the distance between the standardized mean trajectory profiles. This prevented artificial disagreement caused only by permutation of class labels.

Stability was summarized using the proportion of bootstrap samples in which the original three-class solution was reselected, the bootstrap distributions of class proportions, and the adjusted Rand index comparing bootstrap-derived classifications with the original assignments for patients represented in each resample. An adjusted Rand index closer to 1 indicated stronger agreement.

The bootstrap procedure assessed internal reproducibility within the source cohort and was not considered a substitute for external validation.

## S8. Multivariable association models

The primary association between trajectory phenotype and the 90-day composite outcome was estimated using logistic regression. Phenotype 1 served as the reference category.

Model 1 was unadjusted.

Model 2 adjusted for age, sex, body mass index, previous HF hospitalization, left ventricular ejection fraction phenotype, and New York Heart Association functional class.

Model 3 additionally adjusted for diabetes mellitus, coronary artery disease, atrial fibrillation, chronic kidney disease, admission systolic blood pressure, baseline log-transformed NT-proBNP, serum sodium, and discharge use of sodium-glucose cotransporter 2 inhibitors, angiotensin receptor-neprilysin inhibitors or angiotensin-converting enzyme inhibitors/angiotensin receptor blockers, beta-blockers, and mineralocorticoid receptor antagonists.

An additional baseline-biomarker-adjusted model added baseline log-transformed NLR, serum albumin, and eGFR to Model 3 to examine whether the associations were explained by admission levels of the biomarkers used to construct the trajectories.

When exact event dates were available, Cox proportional hazards models were used as supplementary analyses, with time from discharge to the first component event as the outcome. For HF-related readmission evaluated separately, death before HF-related readmission was treated as a competing event using a Fine–Gray subdistribution hazards model.

## S9. Incremental prognostic analyses

The incremental prognostic value of trajectory phenotype was assessed using three nested logistic regression models.

The clinical model included age, sex, left ventricular ejection fraction phenotype, New York Heart Association functional class, previous HF hospitalization, chronic kidney disease, admission systolic blood pressure, and baseline log-transformed NT-proBNP.

The clinical-plus-baseline-biomarker model additionally included baseline log-transformed NLR, serum albumin, and eGFR.

The trajectory-enhanced model further included trajectory phenotype as a categorical variable.

Discrimination was quantified using the area under the receiver operating characteristic curve. Differences in area under the curve between nested models were evaluated using DeLong’s test. Model fit was compared using likelihood-ratio tests. Overall prediction error was assessed using the Brier score, and calibration was examined using calibration plots. Decision-curve analysis was used to explore net benefit across clinically relevant threshold probabilities.

These analyses were performed to quantify incremental prognostic information and were not intended as formal prediction-model development, validation, or model-impact assessment.

## S10. Sensitivity analyses

**SA 1: Complete-case analysis.**
The primary multivariable logistic regression was repeated among patients with complete data for all included covariates.

**SA 2: Multiply imputed analysis.**
The primary model was fitted in each of the 20 imputed datasets, and estimates were pooled using Rubin’s rules.

**SA 3: Disease-specific composite outcome.**
All-cause readmission in the primary composite outcome was replaced by adjudicated HF-related readmission, while all-cause mortality remained part of the composite.

**SA 4: Exclusion of advanced baseline renal dysfunction.**
Patients with baseline eGFR <30 mL/min/1.73 m² were excluded.

**SA 5: Alternative T3 definition.**
The primary trajectory model was refitted after replacing the pre-discharge T3 node with a fixed 96–168-hour post-admission window.

**SA 6: Alternative renal variable.**
Serum creatinine was substituted for eGFR in the multivariate trajectory model.

**SA 7: Inverse probability-of-observation weighting.**
Stabilized inverse probability-of-observation weights were estimated among all 822 clinically eligible discharge survivors with ascertainable outcomes. The probability of inclusion in the complete T1–T3 trajectory cohort was modelled using age, sex, body mass index, previous HF hospitalization, New York Heart Association functional class, left ventricular ejection fraction phenotype, chronic kidney disease, admission systolic blood pressure, baseline NLR, serum albumin, eGFR, serum sodium, and log-transformed NT-proBNP. Weights were truncated at the 1st and 99th percentiles. Covariate balance before and after weighting was evaluated using absolute standardized mean differences, with values <0.10 indicating acceptable balance.

**SA 8: T1–T2-only trajectory classification.**
The multivariate trajectory model was independently refitted using only T1 and T2 measurements among 742 patients with complete biomarker data through 72 hours. Because the model was re-estimated in this larger two-node cohort, the resulting classes were not treated as direct reassignments of the primary T1–T3 phenotypes.

**SA 9: Additional adjustment for in-hospital severity, treatment intensity, and NT-proBNP response.**
Model 3 was additionally adjusted for cumulative intravenous loop-diuretic dose through T3, intravenous vasodilator use, inotrope or vasopressor use, non-invasive ventilation, ICU or coronary care unit care, and percentage change in NT-proBNP from T1 to T3.

**SA 10: Additional adjustment for medication exposure before T3.**
Model 3 was additionally adjusted for sodium-glucose cotransporter 2 inhibitor, glucagon-like peptide-1 receptor agonist, and systemic corticosteroid exposure before T3.

**SA 11: Additional adjustment for baseline trajectory biomarkers.**
Baseline log-transformed NLR, serum albumin, and eGFR were added to Model 3 to determine whether trajectory phenotype associations persisted beyond the admission levels of the biomarkers used for phenotype derivation.

All statistical analyses were performed using R version 4.5.1. Statistical tests were two-sided, and p < 0.05 was considered statistically significant for the primary analyses. Sensitivity analyses were interpreted according to the direction, magnitude, and precision of the estimates rather than solely according to statistical significance.

# References

[1] McDonagh TA, Metra M, Adamo M, et al. 2021 ESC Guidelines for the diagnosis and treatment of acute and chronic heart failure. Eur Heart J. 2021;42(36):3599–3726. doi:10.1093/eurheartj/ehab368.

[2] Heidenreich PA, Bozkurt B, Aguilar D, et al. 2022 AHA/ACC/HFSA Guideline for the Management of Heart Failure: a report of the American College of Cardiology/American Heart Association Joint Committee on Clinical Practice Guidelines. J Am Coll Cardiol. 2022;79(17):e263–e421. doi:10.1016/j.jacc.2021.12.012.

[3] Bozkurt B, Coats AJS, Tsutsui H, et al. Universal Definition and Classification of Heart Failure: a report of the Heart Failure Society of America, Heart Failure Association of the European Society of Cardiology, Japanese Heart Failure Society and Writing Committee of the Universal Definition of Heart Failure. J Card Fail. 2021;27(4):387–413. doi:10.1016/j.cardfail.2021.01.022.

[4] Charlson ME, Pompei P, Ales KL, MacKenzie CR. A new method of classifying prognostic comorbidity in longitudinal studies: development and validation. J Chronic Dis. 1987;40(5):373–383. doi:10.1016/0021-9681(87)90171-8.

[5] Inker LA, Eneanya ND, Coresh J, et al. New creatinine- and cystatin C-based equations to estimate GFR without race. N Engl J Med. 2021;385(19):1737–1749. doi:10.1056/NEJMoa2102953.

[6] Proust-Lima C, Philipps V, Liquet B. Estimation of extended mixed models using latent classes and latent processes: the R package lcmm. J Stat Softw. 2017;78(2):1–56. doi:10.18637/jss.v078.i02.

[7] Nagin DS, Odgers CL. Group-based trajectory modeling in clinical research. Annu Rev Clin Psychol. 2010;6:109–138. doi:10.1146/annurev.clinpsy.121208.131413.

[8] Khwaja A. KDIGO clinical practice guidelines for acute kidney injury. Nephron Clin Pract. 2012;120(4):c179–c184. doi:10.1159/000339789.
